# Characterization and target genes of nine human PRD-like homeobox domain genes expressed exclusively in early embryos

**DOI:** 10.1038/srep28995

**Published:** 2016-07-14

**Authors:** Elo Madissoon, Eeva-Mari Jouhilahti, Liselotte Vesterlund, Virpi Töhönen, Kaarel Krjutškov, Sophie Petropoulous, Elisabet Einarsdottir, Sten Linnarsson, Fredrik Lanner, Robert Månsson, Outi Hovatta, Thomas R. Bürglin, Shintaro Katayama, Juha Kere

**Affiliations:** 1 Biosciences and Nutrition, Karolinska Institutet, Huddinge, Stockholm, Sweden; 2 Competence Centre on Health Technologies, Tartu, Estonia; 3 Department of Clinical Science, Intervention and Technology (CLINTEC), Karolinska Institutet, Huddinge, Stockholm, Sweden; 4 Molecular Neurology Research Program, University of Helsinki and Folkhälsan Institute of Genetics, Helsinki, Finland; 5 Department of Medical Biochemistry and Biophysics, Karolinska Institutet, Stockholm, Sweden; 6 Department of Laboratory Medicine, Division of Clinical Immunology, Karolinska Institutet, Huddinge, Stockholm, Sweden; 7 Department of Biomedicine, University of Basel, Basel, Switzerland

## Abstract

PAIRED (PRD)-like homeobox genes belong to a class of predicted transcription factor genes. Several of these PRD-like homeobox genes have been predicted *in silico* from genomic sequence but until recently had no evidence of transcript expression. We found recently that nine PRD-like homeobox genes, *ARGFX*, *CPHX1*, *CPHX2*, *DPRX*, *DUXA*, *DUXB*, *NOBOX, TPRX1* and *TPRX2*, were expressed in human preimplantation embryos. In the current study we characterized these PRD-like homeobox genes in depth and studied their functions as transcription factors. We cloned multiple transcript variants from human embryos and showed that the expression of these genes is specific to embryos and pluripotent stem cells. Overexpression of the genes in human embryonic stem cells confirmed their roles as transcription factors as either activators (CPHX1, CPHX2, ARGFX) or repressors (DPRX, DUXA, TPRX2) with distinct targets that could be explained by the amino acid sequence in homeodomain. Some PRD-like homeodomain transcription factors had high concordance of target genes and showed enrichment for both developmentally important gene sets and a 36 bp DNA recognition motif implicated in Embryo Genome Activation (EGA). Our data implicate a role for these previously uncharacterized PRD-like homeodomain proteins in the regulation of human embryo genome activation and preimplantation embryo development.

Human preimplantation development, starting with the fusion of egg and sperm and continuing to blastocyst implantation^1^ is characterized by drastic changes in gene expression, especially a massive degradation of oocyte transcripts and a gradual, cascade-like initiation of transcription from the embryo genome^2^,^3^. The cellular factors responsible for the Embryo Genome Activation (EGA) have not been fully characterized. However, recently the PRD-like homeodomain transcription factors were implicated in human EGA and preimplantation development^3^.

The PRD-like homeobox genes form one of several classes of homeobox genes^4^. Homeobox genes are evolutionarily conserved and the majority of these genes are critical for the regulation of development^5^. Examples of homeobox genes include the *HOX* cluster genes^6^ that are known to play key roles in cell fate determination and cell differentiation. Homeobox genes are relatively well characterized in several model organisms, especially in *Drosophila melanogaster*, and several of the PRD-like homeobox genes have previously been suggested to be expressed in the human germline^7^. The PRD-like homeobox genes share a similar homeodomain, but they lack the actual PRD domain^4^. The homeodomain consists of approximately 60 amino acid (aa) residues and folds into a N-terminal arm and three alpha-helices^8^. The N-terminal arm and the third helix have been implicated in DNA-binding specificity and DNA-sequence recognition^9^.

Our recent study on human preimplantation development revealed the expression of 14 PRD-like homeodomain transcription factors during EGA, and we validated the presence of seven factors by cDNA cloning^3^. Most of these transcription factors had been predicted^7^,^10^, but only a few had been validated on transcript level^11^.

Here we describe the molecular cloning of several new splice variants for altogether nine human PRD-like homeobox genes, *ARGFX* (arginine-fifty homeobox; altogether six splice variants), *CPHX1* (cytoplasmic polyadenylated homeobox 1), *CPHX2* (cytoplasmic polyadenylated homeobox 2; three splice variants), *DPRX* (divergent paired-related homeobox; two splice variants), *DUXA* (double homeobox A), *DUXB* (double homeobox B; three splice variants), *TPRX1* (tetra-peptide repeat homeobox 1), *TPRX2* (tetra-peptide repeat homeobox 2; three splice variants) and *NOBOX* (*NOBOX* oogenesis homeobox). We show the first evidence of *TPRX2P* expression and propose that it should be renamed *TPRX2* as previously suggested^7^. Many of these PRD-like homeobox genes appeared primate specific, expressed at very low levels in most tissues and restricted to preimplantation development. Experimental overexpression of the PRD-like homeodomain transcription factors in human embryonic stem cells (hESCs) reveals target genes that intersect with a significant number of the genes first activated in human embryos. The target gene promoters are enriched for the 36 bp motif previously predicted to play a major role in human EGA^3^.

## Results

### Cloning of PRD-like homeobox genes from human 8-cell stage embryos

Our previous transcription start site (TSS) enriched data on human preimplantation embryos suggested unannotated transcripts for *ARGFX, CPHX1, CPHX2, DPRX, DUXA* and *DUXB*^3^. Furthermore, two highly similar Tprx family genes TPRX1 and TPRX2 were predicted. We described previously the cDNA cloning of a major splice form for *ARGFX, CPHX1, CPHX2, DPRX, DUXA* and *DUXB*^3^. In the present study, we cloned novel splice variants of *NOBOX, TPRX1* and *TPRX2* together with additional splice variants for the *ARGFX, CPHX2, DPRX* and *DUXB* genes from human 8-cell embryos.

**Figure 1:**
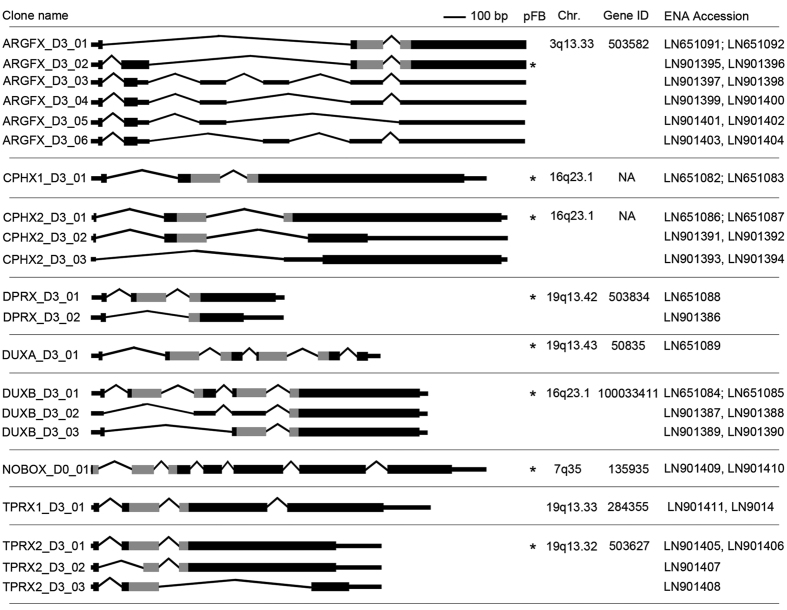
Exon-intron structure of PRD-like homeobox genes. Exons are drawn to scale and represented by horizontal boxes. 5′ and 3′ UTRs are shown as thinner boxes, protein coding region as thicker boxes with homeobox sequence colored grey. Introns are represented by solid lines. Chromosomal locations and Gene IDs, if available, are given on the right. *Indicates the isoform used in the overexpression experiment. NA, not available.

We amplified and cloned transcripts for each gene by PCR from non-sheared full-length cDNA libraries of three whole 8-cell embryos prepared according to the sequencing library preparation protocol by Tang *et al*.^12^. The validated transcripts are visualized in Fig. 1 and in UCSC Genome Browser views in Supplemental Figure S1 together with previously predicted gene sequences and the cloning primer locations. Forward primers were designed on the TSS sequences, and reverse primers by predicted gene structures and low level downstream reads (exon painting). The full gene names, references, identifiers, cloning primers and clone origins are shown in Table 1. The sequences for *ARGFX, DPRX, DUXA* and *DUXB* had been previously suggested^7^,^10^, but only ARGFX had been confirmed experimentally^11^. *NOBOX* was amplified from cDNA that was reverse transcribed from commercially available poly-A enriched human ovary RNA. The partial transcript sequence for *NOBOX* had been previously validated by cDNA cloning^13^, and two additional synthetic constructs are presented in GenBank (BC152834 and AB528776). Current RefSeq annotation follows the structure of the synthetic constructs for exons 1–8 (Supplementary Figure S2). However, our data suggested a novel intronic TSS resulting in a novel transcript with novel first exon, lacking exons number 1–4 and a shortened exon 5 compared to the RefSeq variant (Supplementary Figure S2). The longer exon 5 of the RefSeq variant disrupts the homeodomain, which is encoded by exons 1–3 of our new transcript variant. Our predicted homeodomain structure, however, corresponded to that previously presented^13^.

Three genes, *CPHX1, DUXB* and *CPHX2* form a gene cluster on 16q23.1 within a 68 kb segment on the same strand (chr16:75,693,001–75,761,000) (Supplemental Figure S3). These genes are currently lacking genome annotation, even though they have been predicted in human based on syntenic genomic location between human and mouse^14^. The alignment of amino acids between human *CHPX1* and *CPHX2* confirms high sequence conservation also outside the homeodomain (Supplemental Figure S4a).

*CPHX2* amplification produced three different splice variants, two of which contained exons 1, 2, and variably exon 3, and one splice variant lacking exon 2. Different open reading frames (ORF) were predicted for each three splice variants, with only one having a complete homeodomain (Fig. 1).

The PCR amplification using the *TPRX1* (Gene ID: 284355) TSS as a primer led to the discovery of *TPRX1* itself from one 8-cell embryo, but in addition three additional splice variants of *TPRX2P* from three independent 8-cell embryos. *TPRX2P* is closely related to *TPRX1* located 18.3 kb away at chr19:48,307,772–48,307,835. *TPRX2P* was previously annotated as a pseudogene but proposed to be named *TPRX2* if shown to be expressed^7^. Therefore, we suggest to rename *TPRX2P* as *TPRX2*. All *TPRX2* variants included three exons of variable lengths, but only the longest splice variant encoded a full homeodomain (Fig. 1). The amino acid sequence alignment between *TPRX1* and *TPRX2* showed high similarity of the first 140 amino acids including the homeodomain (Supplementary Figure S4b).

The PCR amplification revealed a single splice variant for *CPHX1, DUXA* and *NOBOX*, all encoding for at least one full homeodomain. Only one of the two validated *DPRX* transcripts encoded for full homeodomain. *ARGFX* amplification gave two splice variants with the predicted ORF consistent with gene model and four additional splice variants (Fig. 1). Amplification of DUXB resulted in three amplicons of which only the longest was predicted to contain both homeodomains.

The most likely functional splice variant containing the full-length homeodomain sequence and observed in all three independent 8-cell embryo libraries is marked in Fig. 1 with an asterisk. These isoforms were subsequently used in the hESC overexpression experiment. The genomic locations of the PRD-like homeobox genes are shown in Supplementary Figure S5.

### Expression of PRD-like homeobox genes in early embryos and pluripotent stem cells

**Figure 2:**
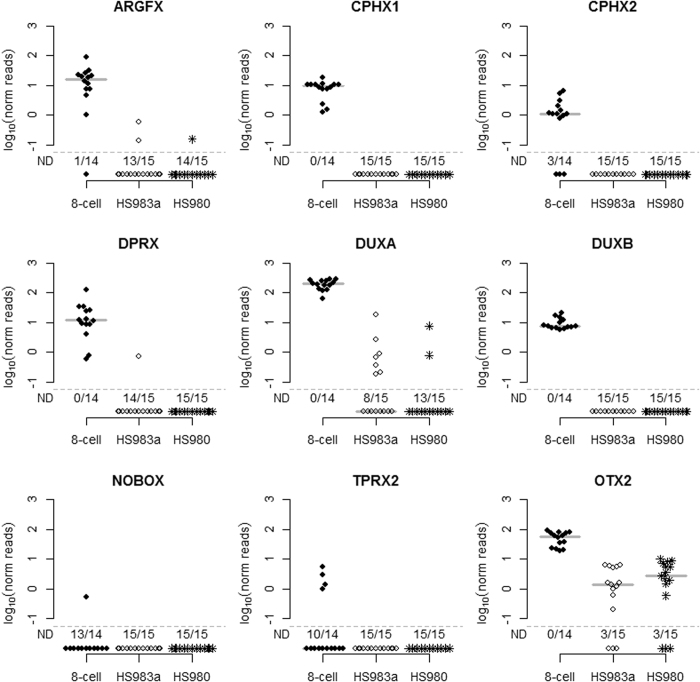
Expression of PRD-like homeobox genes in 8-cell blastomeres and hESCs. STRT RNAseq expression values are shown for two 8-cell stage blastomeres (14 single cells in total) and for two different hESCs (HS980 and HS983a, 15 single cells each) at single cell resolution. Each dot indicates a single cell: 8-cell (filled circle), hES cell line HS983a (not filled circle) or hES cell HS980 (star). Y-axis shows log10 transformed spike-in normalized expression values. Vertical line indicates mean values. ND, not detected, indicates the number of cells in which the gene of interest was not detectable over the total number of cells analyzed with the threshold of one sequencing read per cell.

To investigate the developmental specificity of PRD-like homeobox gene expression, we used single cell RNA sequencing (RNAseq) on single blastomeres from two 8-cell stage embryos and two different hESC lines^15^ and compared the expression patterns (Fig. 2).

In concordance with our previous results^3^, we detected the expression of *ARGFX, CPHX1, CPHX2, DPRX, DUXA, DUXB, NOBOX, TPRX2* and *OTX2* in single blastomeres from 8-cell stage embryos. *OTX2* was included as a positive control gene representing a previously characterized PRD-like homeobox gene. *CPHX1, DPRX, DUXA, DUXB*, and *OTX2* expression was detected in all blastomeres with highest expression levels for *DUXA* and *OTX2*. *ARGFX* and *CPHX2* expression was detected in the majority of blastomeres. *NOBOX* and *TPRX2* expression was detected in one and four single blastomeres, respectively. The largest variation in expression levels among the different blastomeres was detected for *ARGFX* and *DPRX*, whereas *DUXA* and *DUXB* expression was at similar levels for all blastomeres.

In contrast to the expression of PRD-like homeobox genes in blastomeres, the expression in hESCs was much lower. Thus, *CPHX1, CPHX2, DUXB, NOBOX* and *TPRX2* expression was not detected in any of the two different hESC lines (Fig. 2). Both *ARGFX* and *DPRX* expression was detected in only three or one single cell, respectively. Only *DUXA* and *OTX2* were readily detectable in the hESCs, albeit only in a subset of the cells. *DUXA* was detected in seven out of 15 HS983a cells and in two out of 15 HS980 cells. The positive control *OTX2* was detected in 12 out of 15 cells from both hESC lines. The expression levels detected in the hESCs were lower than that for human single blastomeres for all PRD-like homeobox genes detected.

In order to independently confirm the developmental pattern of PRD-like homeobox gene expression, we applied PCR on an additional set of cDNA samples, including HS980 and whole 8-cell stage embryo libraries. In this manner, we detected only low levels of expression of *ARGFX, CPHX1, DPRX, DUXA* and *DUXB* in three hESC lines, including HS980 (Supplementary Figure S6).

### The majority of cell types and tissues lack PRD-like homeobox gene expression

To investigate mRNA expression of the PRD-like homeobox genes in different cell types and tissues, we used the FANTOM5 database^16^,^17^. Only *OTX2* was shown to be expressed in the FANTOM5 database, whereas all the new genes were barely detectable in very few of the 1829 samples (Supplemental Table S1). Specifically, none of the new transcripts had an annotated transcriptional start site (TSS) in FANTOM5 data. Only one unannotated promoter corresponded to one of our genes, namely the *DUXB* TSS, but it lacked correct annotation due to the fact that there was no annotation for *DUXB* itself in FANTOM5.

The lack of expression in FANTOM5 data suggested possible silencing of the genes in later stages of development. To investigate methylation as a possible silencing mechanism of the PRD-like homeobox genes, we considered the DNA methylation status of their promoters in human sperm, preimplantation embryos and embryonic tissues^18^. Percentage of methylated CpG-s from around 1000 bp from the homeobox genes TSS was plotted for all the samples and replicas with sequencing coverage of at least 5x (Supplementary Figure S7). The results show hypomethylation for all the detected homeobox genes in preimplantation embryos compared to various ESC-s and embryonic tissues. This suggests a rapid epigenetic silencing mechanism after the preimplantation development. The more widely expressed control gene OTX2 however was not similarly methylated in course of development. Additionally we assessed the methylation status of these genes in blood cells^19^. The results show that in blood cells these genes were highly methylated, supporting the mechanism of epigenetic silencing in adult tissues (Supplementary Figure S8).

### Overexpression of the PRD-like homeobox genes in hESC identifies target genes

We overexpressed the PRD-like homeobox genes in hESC to identify their target genes. The pFastBac vector used contained both the gene of interest and an eGFP fluorescent marker, enabling separation of transfected cells from non-transfected cells by Fluorescence Activated Cell Sorting (FACS). We could then verify the co-expression of the two proteins in the same cells by the simultaneous expression of eGFP and the red fluorescence gene mCherry control both by microscopy (Supplementary Figure S9a) and by FACS analysis (Supplementary Figure S9b). Successful sorting of the transfected versus non-transfected cells was further confirmed by mapping the sequencing reads to the original pFastBac vector backbone. The vast majority of reads from the GFP positive samples originated from the overexpression vector, while there were very few reads detected in the GFP negative samples (Supplementary Figure S9c).

**Figure 3:**
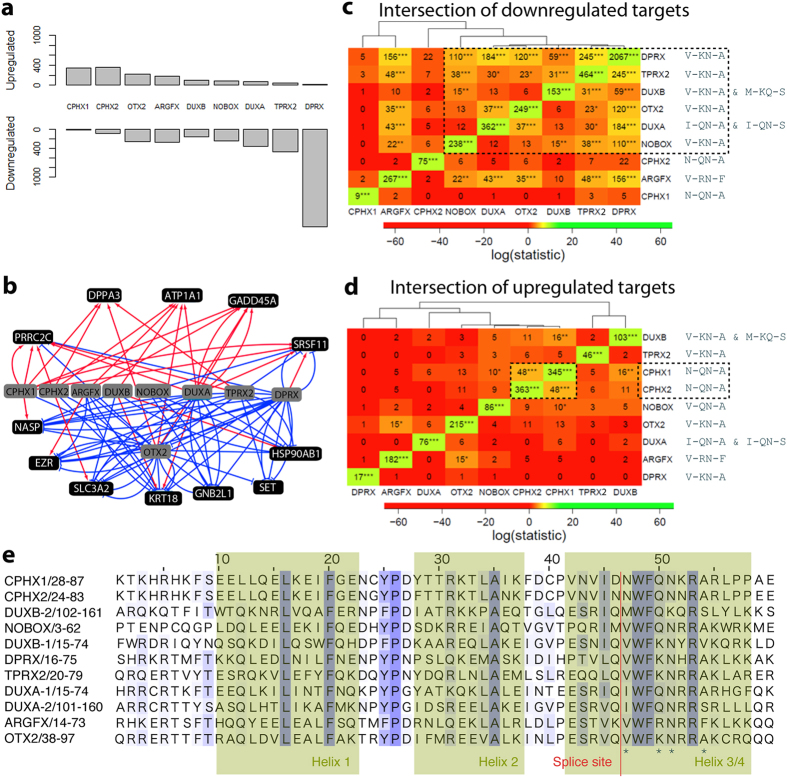
Target genes of the PRD-like homeobox genes in hESCs. The homeobox genes were overexpressed in hESCs followed by transcriptional profiling by RNAseq. (**a)** The number of up- and down-regulated target genes following transfection for 9–11 hours. (**b**) The most commonly occurring up- and down-regulated target genes are shown as a gene network. (**c)** Similarity of the down-regulated target gene lists as observed by performing chi-squared test to the pairwise overlap of all the gene sets. (**d)** Similarity of the up-regulated target genes lists as observed by performing chi-squared test to the pairwise overlap of all the gene sets. The color indicates logarithmic value of the chi-squared test statistics, and the significance of higher than expected overlap is shown by asterisk according to the multiple-testing corrected Fisher’s exact test value (p < 10^−2^*, p < 5 × 10^−5^**, p < 5 × 10^−8^***)**. (e**) Alignment of the amino acid sequences for all homeodomains in the *CPHX1*, *CPHX2*, *ARGFX*, *DUXB*, *NOBOX*, *DUXA*, *TPRX2* and *DPRX* genes. Conserved residues are marked in blue, homeodomain helices have a green background. A conserved splice-site is marked by a red line. *Mark the position of variable amino acids in the DNA-binding domain that are indicated in (**c**,**d**). Dotted line around the amino acid stretch shows grouping of similar genes (**c**,**d**). The red line marks the splice site characteristic for PRD-like homeobox genes^4^,^36^.

After quality control and the exclusion of one disqualified GFP negative control sample, we performed the differential expression analysis as previously described^20^. We listed the differentially expressed TSSs from each comparison at p < 0.05, and for robustness, we considered for further analyses the intersection of lists against three control conditions. The analysis yielded 18 gene sets in total: upregulated and downregulated gene sets for all the PRD-like homeobox genes and the *OTX2* control (Fig. 3a).

Among the regulated target genes we identified 12 common target genes that were either upregulated by at least four or downregulated by at least five of the different PRD-like homeodomain transcription factors. These 12 common target genes included, among others, the cell-cycle gene *NASP*, the pluripotency associated gene *DPPA3*, the trophoblast determining gene *KRT18*, the pre-mRNA processing genes *PRRC2C* and *SRSF11* and the homeodomain gene *OTX2* itself (Fig. 3b).

Furthermore, there were some targets that were specific for a subset of the homeodomain genes. For example, three out of the four Yamanaka factors (*KLF4, MYC, POU5F1, SOX2*) were differentially regulated by *TPRX2, OTX2, DUXA* and *DPRX*: *SOX2* was downregulated by *TPRX2* and *OTX2*, *MYC* was downregulated by *OTX2, DUXA* and *DPRX*, and *POU5F1* was upregulated by *DPRX* and downregulated by *OTX2*. Interestingly, *CPHX1* upregulated *BCAP31* and *USP9X*, two genes important for gamete generation^21^.

### The PRD-like homeodomain transcription factors may act both as activators and repressors

The number of differentially expressed genes varied between the different PRD-like homeodomain transcription factors, with *DPRX* regulating the largest number of genes. The majority of *DPRX* target genes were downregulated and showed overlap with *TPRX2* and *ARGFX* downregulated target genes (Fig. 3c). The smallest number of significantly regulated genes was found for *DUXB*. We found distinct roles for the PRD-like homeodomain genes as either mainly activators, such as *ARGFX, CPHX1* and *CPHX2* (Fig. 3a,c), or as mainly repressors, such as *DPRX, DUXA* and *TPRX2* (Fig. 3a,c). For testing the application of our results to the context of human embryos, we looked at the expression of up-regulated genes by the three activators (*CPHX1, CPHX2* and *ARGFX*) in human embryos^2^. An independent RNA sequencing dataset from human embryos and ESC-s showed up-regulation of the target genes in the course of embryo development for all three activators (Supplementary Figure S10). Full lists of the target genes, their corresponding TSSs and genomic annotations are given in Supplementary Table S2.

To further investigate the target gene profiles, we performed cross-comparisons between all upregulated or downregulated genes. The significance of the overlap was evaluated by Chi-squared test statistic or Fisher’s exact test. A major overlap was found between the upregulated targets of *CPHX1* and *CPHX2* (p < 2 × 10^−14^) (Fig. 3d), and genes upregulated by *DUXB* and *CPHX1* (p < 2 × 10^−8^), *ARGFX* and *OTX2* (p < 2 × 10^−5^), and *CPHX1* and *NOBOX* (p < 2 × 10^−5^).

In the same manner, the comparison between downregulated target genes yielded a number of significant overlaps; the target genes downregulated by *DPRX, TPRX2, DUXB* and *NOBOX* all have significant overlap between each other (Fig. 3c). Furthermore, *DPRX* and *ARGFX* share a significant number of downregulated target genes with all other PRD-like homeodomain genes except for *CPHX1, CPHX2* (both) or *DUXB* (only *ARGFX*). We also tested the overlaps of the target genes upregulated by one factor and downregulated by another (Supplementary Figure S11). A significant overlap was observed between the downregulated targets of *DPRX* and upregulated targets of both *CPHX1* (p < 2 × 10^−8^) and *CPHX2* (p < 2 × 10^−14^). These data suggest tentative regulatory networks controlling human EGA.

### Target gene profiles can be influenced by the amino acid sequence of the homeodomain

Since the members of the PRD-like homeobox gene family share similar amino acid sequences in the homeodomains (Fig. 3e), we hypothesized that they might target the same genes through similar DNA-binding properties. Thus, we correlated the observed overlaps of the target genes with sequence similarities of the homeobox domains (Fig. 3c,d).

*CPHX1* and *CPHX2* shared a large number of upregulated target genes (Fig. 3c), and they had an almost identical homeodomain, with only 4 aa difference (Fig. 3e). *DUXA* and *DUXB* differed from the others in having two homeodomains. Only *DUXB* had the residues V-KN-A, but both *DUXA* and *DUXB* shared a number of target genes with the others (Fig. 3d). *ARGFX* stood out with a different homeodomain sequence and significant overlap between its downregulated target genes and the target genes of *DPRX*, *TPRX2*, *OTX2*, *DUXA* and *NOBOX*. Comparing the overall homeodomain sequence, *ARGFX* shared 35% and 33% aa identity with *DUXA*, whereas the homeodomains in *DUXB* shared 33% and 28% aa identity with *ARGFX*. The same degree of aa identity in the homeodomain (35%) was seen between *ARGFX* and *TPRX2*. This shows that although the similarities in the DNA binding domain indicate similar target gene profiles, the homeodomain similarity was not alone determining the target gene expression profiles.

### Target genes of PRD-like transcription factors are activated during human preimplantation development

**Figure 4:**
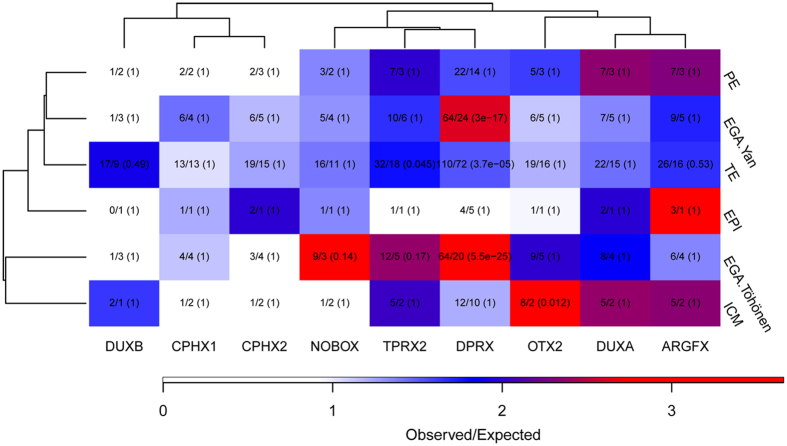
Overlap of target genes with developmentally important gene sets. Chi-squared test was performed for identifying significant number of intersecting genes. Target genes from our overexpression experiment were compared to the developmentally important datasets of genes up-regulated in embryonic genome activation (EGA) (two independent datasets for EGA.Yan^2^ and EGA.Töhönen^3^), epiblast (Epi), Trophectoderm (TE) or inner cell mass (ICM)^22^. The number of observed divided by expected number of intersecting genes is indicated by color scale, and written on the plot followed by multiple-testing corrected p-value in brackets.

We suggested earlier that the new PRD-like homeobox genes might act as key regulators of human early development^3^. In order to further test this hypothesis, we analyzed the overlap between the differentially expressed target genes in our overexpression experiments and the genes upregulated at various stages of preimplantation development: EGA^2^,^3^, trophectoderm (TE), epiblast (EPI), primitive endoderm (PE) and inner cell mass (ICM)^22^ (Fig. 4). The similarity of targets was tested by Chi-squared test after matching the dataset-specific TSSs for EGA genes from Töhönen, *et al*. The lists of upregulated genes from Yan *et al*. and Blakeley *et al*. were calculated as described in materials and methods, and the Chi-squared test was performed by matching the gene names. The number of observed intersecting genes between the experimentally regulated gene sets and the preimplantation gene sets was calculated and compared with the expected number of intersecting genes occurring by chance. We report multiple testing corrected p-values.

Most of the comparisons with PE, ICM and EPI gene sets yielded no statistically significant overlaps with the experimental gene sets, except for OTX2. Eight target genes of OTX2 overlapped with ICM genes, while overlap of only 2 genes was expected (p = 0.012). Larger overlap than expected was noted for DPRX (110/72, p = 3.7 × 10^−5^) and TPRX2 (32/18, p = 0.045) with the TE genes. The most significant overlaps were observed for DPRX with both EGA datasets (Töhönen *et al*.^3^; 64/20, p = 5.5 × 10^−25^ and Yan *et al*.^2^; 64/24, p = 3 × 10^−17^).

### Activity of the homeobox genes via DNA motif implicated in preimplantation development

**Figure 5:**
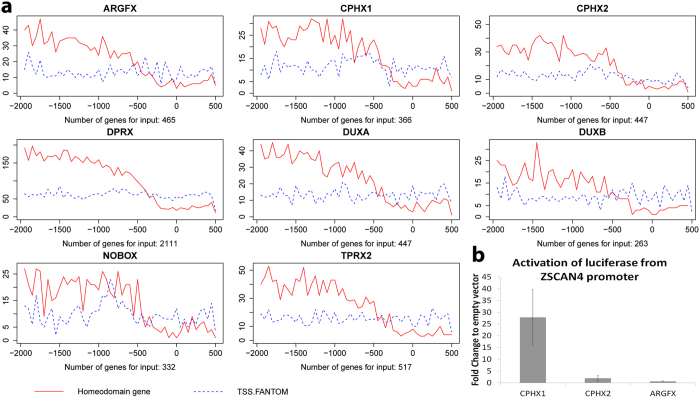
Promoter properties of the homeodomain genes. (**a**) Enrichment of a 36 bp DNA motif in the promotor region (−2,000 ~ + 500 bp distance around the center of the TFE) of the PRD-like homeodomain transcription factor target genes. The motif was enriched upstream of the promoters of genes during human pre-implantation development (red line). The motif is over-represented upstream of the up- and down-regulated genes by homeobox genes and under-represented from about 0 to 500 bp downstream from the TFE position. The motif is not enriched in any specific regions among random start-sites from the FANTOM database (blue dotted line). (**b)** Luciferase expression Fold Change between ZSCAN4 promoter-containing vector and corresponding empty vector (pGL4.25) with transfection by activators CPHX1, CPHX2 and ARGFX in HEK293 cell lines. The average values from three biological replicas are shown, error bars represent standard deviation. The values are normalized to corresponding vector without transcription factor overexpression.

The PRD-like homeobox genes were suggested to act on a motif found enriched in the promoters of EGA genes^3^. In order to test further the hypothesis, we studied the enrichment of the 36 bp embryo motif in the promoters of the experimental target genes (2000 bp upstream and 500 bp downstream from TSS) by the MAST software^23^. The results confirmed the enrichment of the 36 bp motif upstream of the TSS for genes regulated by most of the PRD-like homeobox genes (Fig. 5a). This result further strengthened the notion of the functionality of the predicted motif for PRD-like genes, and supported the 36 bp motif as a key regulatory element in human preimplantation development.

To further investigate the suggested main activators *CPHX1*, *CPHX2* and *ARGFX* as transcription activators, we performed luciferase reporter assays after co-transfecting human embryonic kidney cells (HEK-293) with a transcription factor at a time and a reporter construct with *ZSCAN4* promoter upstream of the luciferase gene. The *ZSCAN4* promoter contained multiple repeats of the 36 bp motif, and *ZSCAN4* was upregulated in EGA^3^. The *ZSCAN4* promoter yielded up to 27 fold increase in luciferase expression when co-transfected with *CPHX1*, but not with CPHX2 or ARGFX, suggesting further specificity of function (Fig. 5b).

## Discussion

The homeodomain proteins make up approximately 15–30% of all animal transcription factors^24^. A number of homeodomain proteins have been shown to be important for early development in vertebrate species^25^,^26^, and some homeodomain proteins are restricted in expression to specific cells at specific developmental stage. In our previous study we detected the expression of PRD-like homeobox genes expressed in oocytes, zygotes or single blastomeres from 4- and 8- cell embryos^3^. The transcript variants of the PRD-like homeobox genes *ARGFX*, *CPHX1*, *CPHX2*, *DPRX*, *DUXA*, *DUXB, TPRX1* and *TPRX2* cloned in this study may be expressed only during a restricted time period, explaining their recent discovery. A thorough investigation of human homeobox loci revealed that *ARGFX, DPRX, TPRX1* and *DUXA* were initially predicted and annotated based on a few existing EST, retrotransposed pseudogene and genomic sequences^7^,^10^. Of the genes presented here only *NOBOX* was previously well characterized and found expressed at high levels in the ovary, specifically in primordial and growing oocytes^27^. Interestingly, our sequencing data indicated a new TSS yielding a novel splice variant of *NOBOX* that we confirmed by cDNA cloning. This novel NOBOX TSS had barely detectable expression in 4- or 8-cell embryos and embryonic stem cells.

In our database searches the PRD-like homeobox genes were undetectable in most cell types and tissues, including the comprehensive FANTOM5 database. In agreement with their absent expression, these genes were rapidly methylated in directly after preimplantation stage in human embryos^18^ , suggesting an epigenetic silencing mechanism (Supplementary Figure S7). This observation was also consistent with the commonly observed silencing of Alu elements enriched in the promoters of the new genes^3^,^28^. During human preimplantation development the DNA methylation levels are reset, which is critical for the normal development^29^,^30^.

Due to ethical reasons, functional studies in human embryos were not possible for these genes. However, we chose the closest and biologically most relevant cell line hESC-s for overexpression experiment. The PRD-like homeobox genes could induce both up- and downregulation of target genes based on the hESC overexpression experiment (Fig. 3a), and the target genes showed significant overlaps both between the different factors themselves (Fig. 3c,d) and between previously published human preimplantation data sets (Fig. 4). While the target genes show expected overlaps with published datasets from embryos, we have to acknowledge that some of the identified target genes might not be the same in human embryos due to the usage of cell model. Since the expression of PRD-like homeodomain transcription factors was very lowly or not at all detected in hESC-s (Supplementary Figure 6), some of the target genes in human embryos might not be overexpressed due to the cellular context such as due to methylation of the gene region.

In the hESCs, *DPRX* was found to downregulate the largest number of target genes (2067), followed by *TPRX2* (464) and *DUXA* (362). We propose that *DPRX* might be needed to restrict the expression of a number of activated genes after the EGA, allowing later lineage specification.

The luciferase reporter assay using a putative *ZSCAN4* promoter placed upstream of the luciferase gene further confirmed that *CPHX1*, but not the other activators *CPHX2* and *ARGFX* acts on *ZSCAN4* promoter (Fig. 5b). Thus the activity of these factors was not determined only by their homeodomain, but rather a combination of homeodomain specificity, possible transactivating domains in the protein or their cellular context and interaction with other proteins. For example, *OTX2* has been shown to interact on protein level with *LHX1* and *FOXA2*^31^. Thus, the cellular context may affect the transcriptional activity of the PRD-like homeodomain proteins.

We studied the possibility that the overlap of target genes for the PRD-like homeobox genes might result from the similarities in the DNA-binding regions of their homeodomains. We observed a large number of genes upregulated by *CPHX1* and *CPHX2*, and they differed in their homeodomain aa sequence at six positions only with a high overall protein sequence similarity (Supplementary Figure S4 a). *ARGFX*, on the other hand, only shared 17 aa identity with the homeodomains of *CPHX1* and *CPHX2*, possibly explaining the low overlap of target genes for these transcription factors even though all three seemed to function mainly as activators (Fig. 3c,d). As an example, *ZSCAN4*, a known inducer of iPS cells^32^ was significantly upregulated by *CPHX1* only. Further experimental evidence is needed in order to define the specificity of the target gene profiles of the homeodomain proteins.

It is known that an aa change at position 52 in the DNA-recognition part of the homeodomain in NOBOX may cause premature ovarian failure due to disruption of the transcription factor binding to the NOBOX-binding consensus sequence^33^. When aligning the different homeodomains for our 9 transcription factors we see that at position 52 the aa varies between the different factors. For example, *ARGFX*, *OTX2*, *TPRX2* and *DUXA* have an arginine residue at position 52, just as *NOBOX*. The overall highly similar *CPXH1* and *CPXH2* differ at this position (lysine or asparagine).

PRD-like transcription factors have been implicated in early development in other organisms such as *Drosophila melanogaster*. For example, the *Drosophila* prd-like homeobox gene Odysseus is involved in germ cell formation^34^. Interestingly, we found that *CPHX1* upregulates the expression of *BCAP31* and *USP9X*, whereas *CPHX2* upregulates *SPIN3*, all three genes involved in human gamete generation according to DAVID annotation. In addition, *BCAP31* is downregulated by *DUXB* and *TPRX2*. Also other germ cell related genes, such as *ZFP42*, *TEX14* and *TFAP2C* were regulated in our overexpression experiment. Targets of the PRD-like homeodomain transcription factors were enriched in genes upregulated during early development (Fig. 4). *OTX2* targets were enriched in ICM, *TPRX2* targets in TE and *DPRX* targets in TE and EGA.

In conclusion, this study presents further evidence of expression for the nine PRD-like genes that we recently identified from human preimplantation embryos^3^. Our data support the hypothesis that the previously uncharacterized PRD-like homeobox genes are specifically expressed during early development, have a key role in the transcriptional dynamics during EGA, and are subsequently silenced as the development progresses from a pluripotent stage towards more highly differentiated stage.

## Materials and Methods

### Human embryos

Human embryos used in this study were collected in Sweden and donated by informed consent by couples who underwent infertility treatment by *in vitro* fertilization (IVF). Cryopreserved cells that were not needed for IVF treatment were donated as an alternative to being destroyed as they had exceeded the maximum legal storage time. Cleavage stage embryos were frozen at the 4-cell stage on day 2 after fertilization. After thawing, embryos were allowed to develop until the 8-cell stage in a sequential culture system (G1/CCM medium, Vitrolife at 37 °C and 5% CO_2_ and 5% O_2_). This study was reviewed and approved by the ethics review boards according to Swedish law (Dnr 2010/937-31/4 of the Regional Ethics Board in Stockholm). All the methods were carried out in accordance with the approved guidelines.

### Library preparation

Human embryo libraries were prepared after thawing the embryos at the 4-cell stage and incubation allowing them to develop until the 8-cell stage. Each single embryo was put into a 0.5 mL PCR tube containing 4.45 μl of freshly prepared cell lysis buffer and the libraries were processed according to the protocol by Tang *et al*.^12^. In total, three 8-cell libraries were prepared.

Human ovary poly-A + RNA was obtained from Clontech (cat# 636152). Human ovary cDNA was prepared using a first strand cDNA synthesis kit (Invitrogen) according to the manufacturer’s instructions.

### PCR primer design

The putative novel transcripts were predicted based on our previous RNAseq data^3^ and these predictions were used for designing PCR cloning primers.

*NOBOX* primers were designed based on an RNAseq peak at FE518599 (chr7:144100745-144100865,-) located in an intron of the human RefSeq sequence NM_001080413.3 (GeneID135935), that was connected to the 3′ UTR of the predicted sequence. The sequences containing ORFs for *CPHX1*, *CPHX2* and *DUXB* were predicted based on FE200101 (chr16:75760315-75760435,-), FE200082 (chr16:75710885-75711065,-) and FE200054 (chr16:75735289-75735420,-), respectively and used for primer design. Predicted sequences used for primer design are provided as Supplemental Text S1. *TPRX1*/*TPRX2* primer design was based on prediction for *TPRX1* with an undefined TSS or TFE at chr19:48,307,772-48,307,835 -, and human RefSeq sequence NM_198479.2 leading to a design of not perfectly matching primers for *TPRX2*. *DUXA*, *DPRX* and *ARGFX* cloning primers were designed based on the human RefSeq NM_001012729.1 (NCBI GeneID 503835), NM_001012728.1 (GeneID 503834) and NM_001012659.1 (GeneID 503582) respectively (Table 1).

### Cloning of ARGFX, CPHX1, CPHX2, DPRX, DUXA, DUXB and TPRX2

cDNA libraries from three single 8-cell embryos were used for cloning of the putative transcripts. The transcripts were amplified from the cDNA libraries using Phusion High-Fidelity DNA polymerase (New England Biolabs) according to the manufacturer’s instructions. For amplification of *DUXA*, *DPRX*, *DUXB*, *TPRX1* and *TPRX2*, the following PCR program was used: 98 °C for 30 s; 40 cycles of 98 °C for 10 s, 65.9 °C (*DUXB*)/67.9 °C (*DPRX*, *TPRX1*,*TPRX2*)/71.5 °C (*DUXA*) for 30 s, 72 °C for 1 min; final extension 72 °C for 10 min. *ARGFX*, *CPHX1* and *CPHX2* were amplified using Touchdown PCR: 98 °C for 30 s; 24 cycles of 98 °C for 10 s, annealing for 30 s, temperature decreasing from 63 °C to 56 °C, 1 °C/3 cycles, 72 °C for 30 s; 16 cycles of 98 °C for 10 s, 55 °C for 30 s, 72 °C for 30 s; final extension 72 °C for 10 min. In order to clone the three differently sized *TPRX2* isoforms, the PCR products were purified from agarose gel using the QIAquick Gel Extraction Kit (Qiagen), and the obtained amplicons were then reamplificated in order to obtain sufficient amount of amplicons for subsequent cloning. PCR products were cloned into the pCR4Blunt-TOPO vector using the Zero Blunt TOPO PCR Cloning kit (Invitrogen) and verified by Sanger sequencing (Eurofins Genomics).

### Cloning of *NOBOX*

Human ovary cDNA was used for cloning putative *NOBOX* transcript. The transcript was amplified from cDNA using HotStarPlus Taq DNA polymerase (Qiagen) according to manufacturer’s instructions. For PCR amplification following steps were applied: 95 °C for 5 min; 40 cycles of 95 °C for 30 s, 60 °C for 30 s, 72 °C for 1 min; final extension 72 °C for 10 min. PCR product was cloned into pCRII-dual promoter TOPO vector using TOPO TA cloning kit (Invitrogen), and sequence was verified by Sanger sequencing (Eurofins Genomics).

### Prediction of ORFs, functional protein domains, and alignment on UCSC genome browser

For open reading frame (ORF) prediction, forward and reverse sequences from each clone were first trimmed for vector sequence using Pregap4 version 1.6-r and then the contig sequences were formed using Gap4 v4.11.2-r (both from the Staden package, http://staden.sourceforge.net/). Mismatching bases were manually checked for quality and edited accordingly. ORFs were predicted and translated using ApE plasmid editor (http://biologylabs.utah.edu/jorgensen/wayned/ape/). Conserved protein domains were predicted applying an NCBI Blastx (http://blast.ncbi.nlm.nih.gov/Blast.cgi) translated nucleotide query on each complete clone sequence against non-redundant protein sequences (nr) and EMBL-EBI InterPro (http://www.ebi.ac.uk/interpro/) on translated sequences. The consensus cDNA and amino acid sequences were aligned by Blat with “psl” as an output (UCSC Genome Browser) and the information was combined to generate a BED file for visualization of custom tracks on a browser (Supplemental Text S2). The alignments were edited manually in order to include short exons.

### Gene expression profiling of hESC lines and single 8-cell blastomeres

A total of 48 single cells were manually picked directly to STRT lysis buffer. The cells included 16 human regular ES cells (HS980), 16 human single-8-cell blastomere derived ES cells (HS983a) and 16 human single blastomeres from 8-cell embryos. The embryos were thawed at 4-cells stage (ThawKit™ Cleave, VitroLife) and cultured in G-1™ Plus media (VitroLife) overnight under standard conditions as performed in the IVF Clinic (5% CO2/5%O2). The zona pellucidae were removed with Tyrode’s solution (Sigma-Aldrich) and embryos were dissociated into single cells using enzymatic (TrypLE, Life technologies) and mechanical separation. The STRT library was prepared according to the modified STRT protocol (Krjutškov, *et al*., accepted) and sequenced on four lanes of Illumina HiSeq 2000 instrument. Pre-processing of STRT reads, quality control and alignments were performed as previously described (Krjutškov, *et al*., accepted). After quality control, 4 outliers (one regular ES cell, one blastomere-derived ES cell and two 8-cell blastomeres) were removed from further analysis due to low RNA content. The gene expression was quantified per transcript far 5′ end (TFE) units^3^. The TFE coordinates for the PRD-like homeobox genes are given in Table 1. The reference file for counting the reads per TFE was defined for PRD-like homeobox genes and spike-in RNAs only. The reads per TFE were counted using SAMtools version 1.1 and htseq-count version 0.6.1, and the data was normalized using external spike-in RNAs as described previously^20^. The raw read counts and normalized expression values for PRD-like homeobox genes and spike-in RNAs used for creating beeswarm plots are given in Supplemental Table S3. The original sequence files as well as aligned BAM-files are accessible from European Nucleotide Archive (ENA) under the accession number PRJEB12467.

### Analysis of methylation pattern for homeobox genes

Publicly available dataset for human sperm, preimplantation embryo, ESC and embryonic tissues was downloaded from GEO (accession number GSE51239, file hDev100bpMeFinal.txt.gz)^18^. 100 bp methylation value percentages were used for genomic positions in the range of 500 bp up- and downstream from the middle of TFE-s for the homeobox genes.

Publicly available dataset for methylation in human blood cells was downloaded^19^. The M-values within the entire homeobox genes’ region was used.

### PCR detection of PRD-like homoebox gene expression

Total RNA extracted from three hESC lines (H9, HS401 and HS980) in three biological replicates were used for cDNA synthesis using SuperScript III First-Strand Synthesis Supermix for qRT-PCR (Invitrogen) according to the manufacturer’s instructions. For PCR we used an input of 22 ng of cDNA and the reactions were done in three biological replicates. As a control, we used 10 ng cDNA from a human 8-cell cDNA library. The PCR reactions were carried out using an ABI PRISM 7500 Fast Real-Time PCR System with FastStart Universal SYBR Green Master mix (Roche) according to the manufacturer’s instructions. The primer sequences are shown in Supplemental Table S4. For validation of the amplicons, the PCR products were cloned into a pCRII-dual promoter TOPO vector using the TOPO TA cloning kit (Invitrogen), and verified by Sanger sequencing (Eurofins Genomics).

### hESC expression vector construction

In order to overexpress *ARGFX*, *CPHX1*, *CPHX2*, *DPRX*, *DUXA*, *DUXB*, *NOBOX*, *OTX2* and *TPRX2* in mammalian cells, the sequences were cloned into a bicistronic pFastBac expression vector. The pFastBac vector was modified as described previously^3^. Briefly, the transcript sequences were amplified from the TOPO vectors using primers containing AscI and PacI restriction sites, digested using AscI and PacI (New England Biolabs) and ligated into pFastBac vector. The primer sequences for cloning the genes in this study are shown in Supplemental Table S5. mCherry fluorescent protein was used as a control, and was amplified from the standard injection marker construct elt-2::GFP for C. elegans (kindly provided by Gert Jansen, The Erasmus University Medical Center, Holland). The final structure of the modified expression pFastBac vector was as follows: CMV enhancer – EF1a promoter – AscI – Gene Of Interest – PacI – IRES – eGFP – WPRE. The IRES (internal ribosomal entry site) element allows for simultaneous expression of two proteins: the inserted gene of interest, and a green fluorescent protein (GFP) marker.

### Overexpression experiments and cell sorting for expression profiling

The hESC line HS401 was cultured on Laminin-521 (Biolamina) in mTeSR™1 media (Stemcell Technologies). One ug of expression vector containing the gene of interest was incubated with 3 μl of Lipofectamine2000 in 50 μl of DMEM. Confluent cells (95–100%) were trypsinized, gently washed with DPBS, and re-suspended in 50 μl of transfection solution. The cell suspension was transferred to a new Laminin-521 coated plate with 50 μl of DMEM and the cells were allowed to settle in the transfection solution for 15 minutes. The media was subsequently changed to mTeSR™1. After 9–11 h of transfection, the cells were washed with DPBS and treated with trypsin to get single-cell solution, washed again with DPBS and suspended in ice-cold DPBS until the analysis. The overexpression time point was chosen by time-course analysis using pFastBac-mCherry vector and further estimation when the first wave of target genes would be expressed. Cell viability was assessed by 1:1000 propidium iodide (Molecular Probes) and cell clusters were removed by FSC and SSC. 75 cells per sample from GFP positive and GFP negative cells were sorted in three replicates into 5 μl of library lysis buffer. The sorting was performed in three replicas from the same transfection well for 75 transfected and 75 non-transfected cells in each replica (altogether 6 samples per gene) for each gene and mCherry control (n = 9), providing two libraries for pooled sequencing of 54 samples by modified STRT protocol (Krjutškov, *et al*. accepted).

The homeobox genes were distributed between two libraries that were processed simultaneously. The first of these, Library 3, included *DUXA*, *DPRX*, *ARGFX*, *OTX2* and mCherry. The second library, Library 4, included *CPHX1*, *CPHX2*, *DUXB*, *TPRX1*, *NOBOX* and mCherry.

### STRT RNAseq analysis for target gene detection

The STRT data was analyzed as described previously^3^. Briefly, the reads were filtered, de-multiplexed by barcodes, joined for the same unique molecular identifiers (UMI), trimmed for the barcodes and UMI-s, and mapped to the human UCSC genome hg19 by TopHat. Outliers were identified by analysis of distance correlation dendrograms, which indicated two outliers for one library (Library 3, Supplementary Figure S12a) and no outliers for the second library (Library 4, Supplementary Figure S12b). The outlier E8 in Library 3 (mCherry, GFP negative) was removed, resulting in one library having one less GFP negative control. The principal components one and two separated two different *DPRX* wells from the controls, and the components 3 and 4 separated none, therefore *DPRX* was not removed.

The signal of STRT sequencing forms clusters that were identified as Transcript Far 5′ End (TFE) as described previously (Krjutškov, *et al*. accepted). Briefly, the aligned BAM files were assembled into putative transcripts by sample types using Cufflinks, followed by extraction of only the 5′-end exons by UCSC tools and followed by merging reads from various sample types by BEDTools. The reads were counted on the identified TFE-s by htseq.

The raw reads were aligned by TopHat to the modified pFastBac backbone vector, and reads counted by htseq, in order to confirm overexpression in the GFP positive versus GFP negative cells.

Three sets of controls were used for performing differential analysis: only mCherry control GFP positive wells (n = 3), only GFP-negative wells from the whole library (n = 22 or n = 23), and the combination of both mCherry control and homeobox specific GFP-negative wells (n = 3 + 3). Estimates of differential expression for the TFE-s were obtained using the R package SAMstrt with positive samples compared to each of the three control (FDR < 0.05 unless indicated otherwise)^20^. Only intersection of the differentially expressed TFE-s from three analysis was used in the study. The identifier TFE-s were mapped to the genome and annotated for genes and genomic regions as described previously^3^. Only the reads mapping to 5′ UTR of coding genes were used in the study.

The original sequence files as well as aligned BAM-files are accessible from ENA by the accession number PRJEB12453.

### Gene expression analysis for public preimplantation datasets

Three publicly available datasets were used for gaining differentially expressed genes lists. Two independent studies were used for gaining the EGA genes lists^2^,^3^. The TFE identifiers up-regulated by either 4- or 8-cell stage were gained from Töhönen *et al*. and the identifiers were matched with at least 1 bp overlap by BEDTools.

The RPKM values were downloaded from Yan *et al*. and up-regulated genes by either 4- or 8-cell stage were calculated by students t-test (FDR < 0.05). Only gene names that had a match in our dataset were used for the analysis. Average RPKM values in Oocytes, Zygotes, 2-, 4-, 8-cell embryos, Morluae, Epiblast and passage 0 ESC-s were used for calculating the gene expression levels of up-regulated target genes.

Raw count values were downloaded from a study that performed RNA sequencing on different lineages of human blastocysts^22^. Students t-test was done for four comparisons in order to obtain genes specifically expressed in TE (TE vs EPI), ICM (EPI vs TE), PE (PE vs EPI) and EPI (EPI vs PE). Ensembl ID-s for the differentially expressed genes (p < 0.05) were converted to the associated gene names by Ensembl Biomart tool^35^ and intersecting gene names with our dataset were used.

The number of intersecting identifiers present in both datasets, not present in either one or both of the datasets were calculated for the Chi-squared test.

### Motif analysis

The MAST tool^23^ was used to search for significant sequence patterns to all given motifs in a gene set. Sequences −2000 and +500 bp from the center of each TFE for differentially regulated genes (FDR < 0.1) were analyzed. A list of all transcription start sites was gained from FANTOM database, and used as a control set to the THE-s.

### Luciferase reporter assay

HEK-293 cells (ATCC) were seeded on 48-well plates in DMEM containing 1 g/l glucose, L-glutamine, pyruvate and supplemented with 10% FBS and 2 mM L-glutamine (all from Gibco). Cells were grown overnight at 37 °C in 5% CO_2_ and transfected with the *ZSCAN4* promoter in pGL4.25 reporter vector described previously^3^ or pGL4.25 luciferase vector (Promega) in combination with *CPHX1*, *CPHX2* or *ARGFX*, all cloned into pFastBac vector as described above. Renilla luciferase vector pGL4.74 [hRluc/TK] (Promega) was co-transfected with other constructs to enable normalization. The concentrations for single constructs were as follows: Luciferase vector 100 ng/well, pFastBac vector 100 ng/well and Renilla luciferase vector 10 ng/well. The transfections were performed using Lipofectamine 2000 (Invitrogen) 0.5 μL/well according to manufacturer’s instructions. Cells were incubated at 37 °C in 5% CO2, harvested 24 h after transfection and subjected to Dual luciferase assay (Promega) in three biological replicates with two technical replicates each, according to manufacturer’s protocol. Luciferase signals were measured using a TECAN infinite M200 (Tecan, Männedorf, Switzerland).

### Data availability statement

All the novel homeobox genes clone sequences were submitted to EMBL, and their ENA accession numbers are given in Fig. 1. The short read sequences and aligned BAM-files from the RNA-seq experiments for the overexpression in ES-cells are uploaded in ENA with the accession number PRJEB12453. The RNA-seq dataset from single-cell hESC-s and 8-cell human embryo have the accession number PRJEB12467.

## Additional Information

**How to cite this article**: Madissoon, E. *et al*. Characterization and target genes of nine human PRD-like homeobox domain genes expressed exclusively in early embryos. *Sci. Rep*. **6**, 28995; doi: 10.1038/srep28995 (2016).

## Supplementary Material

Supplementary Information

Supplementary Dataset 1

Supplementary Dataset 2

## Figures and Tables

**Table 1:** Cloning of PRD-like homeobox genes.

Gene name	Full name & first reference	Gene ID	TFE ID*	TFE Region	Strand	Forward/Reverse Primer	Clone origin
*ARGFX*	arginine-fifty homeobox^7^	503582	FE367949	chr3:121286750–121286860	+	GAGAGACACACCACGTAGGAC**/**TCAGAGAAATCCCAAGTCTACC	8-cell embryo
*CPHX1*	cytoplasmic poyadenylated homeobox 1^14^		FE200101	chr16:75760315–75760435	−	TCTCAGTTGCTTGCTGGTCTC**/**CCTGACCTGCGACTGTGTTT	8-cell embryo
*CPHX2*	cytoplasmic poyadenylated homeobox 2^14^		FE200054	chr16:75710885–75711065	−	GAGTTTCGACATGTCTTCCCAAG**/**TGACCTGTGGCTATGGTTCTGT	8-cell embryo
*DPRX*	divergent paired-related homeobox^7^	503834	FE273734	chr19:54135194–54135433	+	TATCCCTGGACCTGAACCCA**/**ACACATCATTACAACATGTGACT	8-cell embryo
*DUXA*	double homeobox A^7^	503835	FE262918	chr19:57678787–57678897	−	GCTCAGCCTTCAGGACTCTCT**/**GTGAGACAGATTTGGGGTCCA	8-cell embryo
*DUXB*	double homeobox B^10^	100033411	FE200082	chr16:75735289–75735420	−	TTACTCGCTGATCTCCGTGG**/**TCAGCTGAGTGTGCCTACTG	8-cell embryo
*NOBOX*	NOBOX oogenesis homeobox^13^,^27^	135935	FE518599	chr7:144100745–144100865	−	ATGGAACCCACAGAGAATCC**/**ACAAGACGAGCCTACACAGG	Ovary
*TPRX1*	Tetra-peptide repeat homeobox 1^7^	284355		chr19:48307780–48307850	−	TCAGGACTCAGGATGCAAGAC**/**ATCCCAGCAGAGAAACGCTC	8-cell embryo
*TPRX2*	tetra-peptide repeat homeobox 2^7^	503627		chr19:48362000–48363000	+	TCAGGACTCAGGATGCAAGAC**/**ATCCCAGCAGAGAAACGCTC	8-cell embryo

^*^TFE, Transcript far 5′ end, TFE ID refers to TSS data by Töhönen&Katayama, *et al*.^13^.
