# Corrigendum: Characterization and target genes of nine human PRD-like homeobox domain genes expressed exclusively in early embryos

**DOI:** 10.1038/srep32053

**Published:** 2016-09-02

**Authors:** Elo Madissoon, Eeva-Mari Jouhilahti, Liselotte Vesterlund, Virpi Töhönen, Kaarel Krjutškov, Sophie Petropoulos, Elisabet Einarsdottir, Sten Linnarsson, Fredrik Lanner, Robert Månsson, Outi Hovatta, Thomas R. Bürglin, Shintaro Katayama, Juha Kere

Scientific Reports 6: Article number: 2899510.1038/srep28995; published online: 07 14 2016; updated: 09 02 2016

The original version of this Article contained a typographical error in the spelling of the author Sophie Petropoulos, which was incorrectly given as Sophie Petropoulous. This has now been corrected in the PDF and HTML versions of the Article.

