# Supplementary material for: Characterization and target genes of nine human PRD-like homeobox domain genes expressed exclusively in early embryos
Source: Sci Rep. 2016 Jul 14;6:28995. doi: 10.1038/srep28995 (PMC4944136; doi:10.1038/srep28995)

Characterization and target genes of nine human PRD-like homeobox domain genes expressed exclusively in early embryos

Elo Madissoon[1,¶], Eeva-Mari Jouhilahti[1,¶], Liselotte Vesterlund[1], Virpi Töhönen[1], Kaarel Krjutškov[1,2], Sophie Petropoulous[3], Elisabet Einarsdottir[1,4], Sten Linnarsson[3], Fredrik Lanner[5], Robert Månsson[6], Outi Hovatta[5], Thomas R. Bürglin[1,8], Shintaro Katayama[1,7] and Juha Kere[1,4*]

[1] Biosciences and Nutrition, Karolinska Institutet, Huddinge, Stockholm, Sweden

[2] Competence Centre on Health Technologies, Tartu, Estonia

[3] Department of Medical Biochemistry and Biophysics, Karolinska Institutet, Stockholm, Sweden

[4] Molecular Neurology Research Program, University of Helsinki and Folkhälsan Institute of Genetics, Helsinki, Finland

[5] Department of Clinical Science, Intervention and Technology (CLINTEC), Karolinska Institutet, Huddinge, Stockholm, Sweden

[6] Department of Laboratory Medicine, Division of Clinical Immunology, Karolinska Institutet, Huddinge, Stockholm, Sweden

[7] Science for Life Laboratory, Stockholm, Sweden

[8] Department of Biomedicine, University of Basel, Basel, Switzerland

* Corresponding author, E-mail: juha.kere@ki.se

¶ These authors contributed equally to this work.

Supporting Information

Supplemental Text S1. Predicted sequences for PRD-like gene cloning primer design

Supplemental Text S2. UCSC genome browser tracks for novel PRD-like homeobox genes

Supplemental Table S1. PRD-like homeobox gene expression in the FANTOM5 comprehensive promoter database. The number of predicted promoters, normalized tagclusters in predicted transcript or in the whole region of the gene are shown. Values near or below 1 may be considered noise, explaining the lack of predicted promoters for very low expression values. Counts of tagclusters from the FANTOM5 promoter database correlate with the expression of the genes.

Supplemental Table S2. The up- and downregulated gene sets and corresponding TFEs and genomic annotations. All the detected up- and downregulated target genes are shown on separate sheets for each homeodomain transcription factor. The down-regulated and up-regulated target genes are sorted according to the differential expression score from lowest to highest. TFE-s (TFE), gene names (Gene.Name), differential expression score (Score.d.), q-value (q.value...), fold change (Fold.Change), TFE genomic annotation location (annotation), and differential expression direction (regulated) are shown. The column Expressed.in.preimplantation shows whether the gene was detected (average RPKM values over oocytes, zygotes, 2-, 4-, 8-cell embryos, morulaes and blastocysts > 1) or not detected (average RPKM < 1) in Yan, et al., 2013 dataset.

Supplemental Table S3. Expression values for PRD-like homeobox genes in hESC and 8-cell blastomeres

Supplemental Table S4. Primers used for PCR

Supplemental Table S5. Cloning primers for FastBac vector constructs

Supplemental Figure S1. UCSC genome browser image showing gene structure, TFE peaks and transcript prediction. UCSC genome browser view of validated PRD-like transcripts. Primer locations in relation to the previously predicted gene sequences and our validated transcripts are shown. Expression in three different stages of human pre-implantation development, 8-cell, 4-cell and oocyte are visualized below each transcript.

Supplemental Figure S2. NOBOX structure and novel TSS

Supplemental Figure S3. Visualization of *CPHX1-DUXB-CPHX2* cluster at chr16:75,693,001-75,761,000 where all three genes are transcribed from the reverse strand.

Supplemental Figure S4. Similarities in protein sequence inside and outside of the homeodomain. Alignment of a) CPHX1 and CPHX2 and b) TPRX1 and TPRX2 shows protein sequence similarities outside of the highly conserved homeodomain.

Supplemental Figure S5. Chromosome ideogram visualizing the genomic locations of the PRD-like homeobox genes. The clustering of DUXA, DPRX, TPRX2 and TPRX1 on chromosome 19 is shown, with the position of CRX given as well. There is a second cluster of genes on chromosome 16, namely CPHX1, DUXB and CPHX2, whereas TPRXL, ARGFX, NOBOX and OTX2 are located outside of these 2 clusters on chromosome 3, 7 and 14 respectively.

Supplemental Figure S6. Expression of homeobox genes in hESCs and 8-cell embryo. PCR was performed on tree different hESC lines Hs401, H9, HS980 and 8-cell blastomeres. Expression values were obtained by normalizing to GAPDH expression by the formula y= (Ct(*GAPDH*) -

Ct(gene)) + 25. The average of three replicas is shown with standard deviation represented by error bars. A Ct value of 40 was used for not detected samples.

Supplemental Figure S7. The methylation patterns of the PRD-like homeobox genes in human sperm, preimplantation embryo, ESC and embryonic tissue cells. The 100 bp methylation value percentages were plotted for sites within 500 bp up- and downstream of the TFE for the specific homeobox gene. Only sites with at least 5 x coverage were used. Circles indicate each data-point and nothing is plotted when data is missing. Color of the boxplot indicates sample groups: red – sperm; green – 8-cell preimplantation embryo (8-cell); dark blue – inner cell mass of the preimplantation embryo (ICM); light blue – trophectoderm of the preimplantation embryo (TE); purple – blastocyst of the preimplantation embryo (Blast, BlastSingle); yellow – embryonic stem cells; grey – fetal heart and lung tissues.

Supplemental Figure S8. The methylation patterns of the PRD-like homeobox genes in blood cells. The M-values for CpG sites surrounding the homeobox genes ARGFX, DPRX, DUXA, NOBOX and the control gene OTX2 are shown for human blood cells from Infinium Human Methylation 450K bead chip array. The values below 0 indicate hypomethylation, the values above 0 indicate hypomethylation. Values are indicated by physical distance from the TSS of every gene for up to 1500 bp upstream (TSS1500) throughout the 5' UTR, 1-st Exon, Gene Body and 3'UTR.

Supplemental Figure S9. The pFastBac vector construct is functional in hESCs. mCherry red fluorescent protein was cloned into the pFastBac vector in the same manner as the homeobox genes. Both the microscopy images with bright field in top panel, followed by mCherry and eGFP channels (a) and FACS analysis (b) show simultaneous expression of both eGFP and mCherry proteins in the same cell (boxed). Multiple sequence reads from GFP positive samples align to the overexpression vector pFastBac backbone sequence while almost no reads align to the backbone from the GFP negative samples in library 4 (c).

Supplemental Figure S10. Expression of up-regulated gene sets in embryos. Gene expression values for up-regulated target genes by three activators (*CPHX1*, *CPHX2* and *ARGFX*) were studied in human embryo development time-course by Yan, et al, 2013. Average log(RPKM) values for all the up-regulated target gene sets in different biological samples are shown as line-plots for *ARGFX* (red), *CPHX1* (green) and *CPHX2* (blue). Error bars show standard error of the target genes.

Supplemental Figure S11. PRD-like homeodomain transcription factors have common target genes. Similarity of the homeobox target genes was observed by performing a chi-squared test of all pairwise comparisons for both up- and downregulated genes. The number of intersecting genes is shown, as well as a multiple testing corrected p-value associated with the chi-squared test. $p < 10^{-2}$*, $p < 5 \times 10^{-5}$**, $p < 5 \times 10^{-8}$***.

Supplemental Figure S12. Hierarchical clustering and outlier detection. Hierarchical clustering reveals outliers in library 3 (a): C5 and E8. There are no evident outliers in library 4 (b).

Supplementary text 1

Predicted sequences for PRD-like gene cloning primer design

Coordinates refer to Genome assembly GRCh37/hg19

Forward primers were designed within predicted the 5' UTRs, and reverse primer within the predicted 3'UTRs.

Coordinates for predicted 5'UTRs for genes with previous RefSeq predictions:
DUXA (FE262918 chr19:57678787-57678897 -)
DPRX (FE273734 chr19:54135194-54135433 +)
ARGFX (FE367949 chr3:121286750-121286860 +)
TPRX1 (undefined chr19:48,307,772-48,307,835 -), led amplification of **TPRX1** and **TPRX2**

Predicted sequences for PRD-like genes without previous RefSeq predictions:

DUXB (FE200082 chr16:75735289-75735420 -)

>FE200082.Duxb.5'UTR, 41 bp
TTACTCGCTGATCTCCGTGGAGTCCAGGTCTTCTGCCCAAG

>FE200082. Duxb.CDS, 1038 bp
ATGAATTTGGAGGGCACTTCAGGTGGCATACTTCAAAAAGAATTCTGGAGAAACAGAA
TTCAGTATAACCAGAGTCAAAAGGATATCCTCCAATCATGGTTTCAACATGACCCTTTC
CCTGATAAAGCTGCCAGAGAACAACTGGCCAAAGAAATTGGGGTTCCAGAATCTAATA
TTCAGGTTTGGTTTAAAAATTACAGAGTAAAACAGAGAAAACTGGATTATAAGTGCTT
CTCAGAAAAAGATCAAACCCAGGGGCATGACCAGTCCCAGCATCTGACTCAAGAATAC
TTACCTAAAGAAGCCCGACAAAAACAGACATTCATCACATGGACTCAAAAAAACAGG
CTAGTGCAAGCCTTTGAGAGGAACCCATTCCCTGATATTGCTACCAGAAAAAAACTGG
CTGAACAAACAGGCCTGCAGGAATCAAGAATTCAAATGTGGTTTCAGAAACAAAGATC
TCTGTACCTCAAGAAGAGCAGAATGGAGCCCATGAATTTATTGGTAGACGACCCAAAT
GAGAGACCAGATGCAACTGTTGGGTGGCATCCAATCAACCTGTTCCTCCCCACAGACA
GCTCTCATTATTTTTCTTGCTCACATTCTTCCAGCGGGCATGAAACTCTTCCACCTGTTC
TTCCTTCAACCCAGGCTCCTTGGGATCCCTTCAGGTTCCATGTGAGCCAAGGACCAAAT
GTCATGATCATGCAGCCCACACAGGCTGTGCAGGAAGGAGAGAAGTCTGATCAGCCTC
TGATAATTCCGAATCACCTCCTGACACTGCCAATTCTGACAAAGGACTTAGATACTCCG
ACTCCCTTCTGGCTCCAATACCAAGAAGAACACCAAAATCACAAAGAACACTCTGGCT
CGGGAGTACCACAGGTCAAGAGCCATTCTCAGCCTGAACCTGAGCACAGGGAGCAAC
AACCTCTAAATCTGGGTCAGTTTGACATATCGAACATTTTGCAAAGGTGGGACGAGAT
CTGCCAGGCTCTGCTTGCTGAATGGGACCCTCTCAAAGGGACACACTGA

>FE200082. Duxb.3'UTR, 1177bp
GACAGACTTGTGGCAGCAGTAGGCACACTCAGCTGAGAAACATGTCATCCACTTGGCT
AATTGTCCCAGCAATATGTGGAAACCTCAAGCCTTTAGGTCAACTCCTGTAATGCAGA
GTTCTCCAGTAAAAGGTACAACTTggccgggcgcagtggctcacgcctataatcccagcactttgggaggccaaggcg
ggcagatcacgaggtcaggagtttgagaccagcctggccaacatggtgaaaccctgcctctactaaaaatacaaaaattggctgggcgtggtg

gcgcgcgcctgtagtcccagctacccaggaggctaaggcacgagaatcgcttgaacctgggaggcggaggtagcagtgagccgagatcac
gccactgcactccaccctggtgacagagtgagactccgtctcaaaaaaataaataaataaaTAAGTACAACTTGGCAATTTC
tgacatatagaaattggaagtgaggtatggatggctggattggttacagcttggtgtatgccttatttgaacagagtttgaacactcagcagtgtatg
agtggttgaagtatggccactggaattgtccaagacttagctactgttacatatacatactactaagttaggttttcaattttgtctattaagctaggtta
cagttcatccacaagtactcaaatatagaagtacagagtccttctcaggccacatttactttgctttcacatgggttagcatccgagatagagtgattt
tagcctccacaGGATGTATGTGGGGTTTACTTTGGTACAATAAAAATATCTTTGAAAAATAttctt
tttttttttttgagacggagtctcgctctatcgcccaggctggagtgcgatggtgcaatctcggctcactgcaacctctgcctcccggattcaagcga
ttctcctgcctcagcctcctgagttactgggattataggcgtgtgccaccatgcctggctaattttgtatttttagtagagacggggtttcaccatgtt
ggtcaggctggtctcaaactcctgacctcatgatccgcccatctcagcctcccaaagtgctgggattacaggtgtaagccaccatgcccagccT
TGAAAAATATTACTTGTTTATCTTATCCTTTTGAATTCTGTTCCATTATACAATACTTTC
AA

NOTE: DUXB 3’-UTR contains Alu and ERV (small letters), which was masked for the design

CPHX1 (FE200101 chr16:75760315-75760435,-)

>FE200101.Cphx1.5'UTR, 60 bp
GTTGCTTGCTGATCTCAGTTGCTTGCTGGTCTCCGTGAAGTCCAGGTCTTCTACCCCAA
G

>FE200101.Cphx1.CDS, 1218 bp
ATGAATTTGGACGGCACTTCAGGTGGTTTCCCAGCTGAAGAGGATCATCATAATGAAG
AAAGACAAACAAAGAATAAAAGAAAAACAAAACACCGACATAAATTTTCTGAAGAAT
TACTGCAGGAACTTAAGGAAATATTTGGAGAGAACTGTTATCCCGATTACACAACTAG
GAAAACACTGGCCATCAAATTTGATTGTCCGGTAAATGTGATAGATAATTGGTTCCAG
AATAAAAGAGCCAGACTTCCACCTGCAGAAAGACGCAGAATATTTGTTCTTCAGAAAA
AGCATGATTTTCCAGTCCAAGCCCATTCATTTTTAAGCTGCCAGGAGACCCAGGCTGCA
GCTCACAACTATGCCACCAAGCAGAGCCTCTCTGGTGCCCAGAGGGCTCTGATGAGAA
GAGCTGGTTGCTCCCATCTGGAGAAACAGTGGATTCCCAGTCAAGAAATGGGCTATAA
TTGTTTCTCTTTGGAGAACCAAGAGACTCCCAGTCAACAGGTGGGCCCCCAGTGCTCTT
ATCTGGAGAAACCAGGGATTCCCAGTCAACAGGTGGGTTCCCAGTGCTCCTATCTGGA
GAAACTAGGGATTCCCAGTCAACAGGTGGCCTCCCAGAGTTCCTATCTGGTCACAGGC
ACTGAAAAGCATCCAGGCTGTGCTATGGGGTATGGAGGTGACACAGGAAGTGGGCATT
CTGGAAGTGGGCATTCTACTGCCTATCATTTTCTCAGCTACAACTCTGCAGAATGCCTT
CATCCTCCCCCATCTTCTGTGCCATATTTTCATGGAGAAAGGACTGAAACCAAGGAAA
GCCAGCATGCAAGTCCTTTCCTTTTGGATTACGCTCAAGGTGCATATGGGGTGAAGAA
AGACCATTGTCTTTGCTCATTCTGTCTCTCACTGCTGGGACAACAGCAGCAGAATGATT
GGCAGTATCACCTGCAGCAGCACCAACAGCCTCAGAATTACTTAGAGGGGATGATGTT
GCAGGAACAGCTCCCAATGGACTCGGGTCCTTGGGATCTAGGGAAGCAGTGGTCCTCG
GCTCAGTCACAGCTGCAGAGTCAACTGCCTCAGAATAATGGAAAGCCGTTGTGCTCTC
AACTGCAGCACATGTCTCTCCAAATAGCTGCCGACTCACCCCTTCTGCCTCTGGGGCAA
GATATGCAGGAAAGGGCTTCAGAGCAACCCAGGACCCAAATGCAGCAACTTTAA

>FE200101.Cphx1.3'UTR

GTTGAAGAGTGGACAAGGGTCTGCAGCAGGGAACCAAGGATGCAACTCTGCAAAGTA
GTGCTAAGTGTCAGAGAAACACAGTCGCAGGTCAGGCCCACAGTCACCCACACAGTAG
CACAAGGCAGAGCCCTTACCAGAAGCCTCCACATTAAGAAAAGAAAGAAAGAAAGAA
AGAAAAAAGCCAGGTGACATTGTTTGAGCCCAGGAGTTCAAGAAAAGCCTGGGCAAC
ATGGTGAGTCCCTGTCTCTATAAAAAAACTTTTTAAAAAATTAGCTGGGCATGATGGTG
TGCTCCTGTGGTCCCAGCTACTCAGG

CPHX2 (FE200082 chr16:75710885-75711065,-)

> FE200082.Cphx2.5UTR, 41 bp
TTATTTTTTCAGTTGGAAAGGCCCTGGACTGGAGTTTCGAC

> FE200082.Cphx2.CDS

ATGTCTTCCCAAGCTTTCCCAGCTGAAGAGGATCATCATAATGAAGAAAGACAAACAA
AGAAAAAAAGAAAAACAAAACACCGACATAAATTTTCTGAAGAATTATTGCAGGAAC
TTAAGGAAATATTTGGAGAGAACGGTTATCCTGATTTCACCACTAGGAAAACACTGGC
CAACAAATTTGATTGTCCGGTTAACGTGATAAACAACTGGTTCCAGAACAATAGAGCC
AGACTTCCACCGGAAGAAAGACAAAGAATATTTCTTACTTGGAAAAAACACGATTTCC
CAGTCCAAGCCTGTCCATTTTTAAGCCTCCAGGAAACCCAGGCTGCAGCTTCCAACTAT
GCCACAGAGCAGAGTTTTTCCTGTGCCAAGAGGGCTCTGATGAGAAGACCTGGTTGCT
CCCTTCTGGAGAAACAGAGGATTGCCTGTCAACAGATGGGCTACAATTGCTTCTCTTTG
GAGAACCAAGAGACTCCCAGTCAACAGGTGGGCTCCATGTGCTCTTCTCTGGAGAAAC
AAGGGATTCCCAGTCAACAGGTGGGTTCCCAGTGCTCCTATCTGGTCGCAGGTACTGA
AAAGCATCCAGGCTATGCTTTGGAGTATGGAGGTGACACAGGAAGTGAGCATTCTACT
GCCTATCGTTTTCTCAGCTACAACTCTGCAGAATGCCTTCATCCTCCCCCATCTTCTGTG
CCATATTTTCATGGAGAAAGGACTGAAACCAGGGAAAGCCAGCATGCAAGTCCCTTCC
TTTTGGATTACGCTCAAGGTGCTTACGGGGTGAAGAAAGACCATTGTCTTTGCTCATTC
TGTCTCTCACTGCTGCAAGAACAGCAGCAGAATGATTGGCAGTATCACCCACAGCAGC
ACCAACAGCCTCAGAATTACTCAGAGGGGATGATGTTGCAGGAACAGCTGCCAATGGA
CTCGGGTCCTTGGGATCTAGAGAAGCAGTGGCCCTCGGCTCAGTCACAGCTGCAGAGT
CAACTGCCTCAGAATAATGGAAAGCCGTTGTGCTCTCAACTGCAGCACGTGCCTCCCC
AAATAGCTGCCAACTCACCCCTGCTGCCTCTGGGGCAAGATATGCAGGTAGGGGCTTC
AAGCAACTCAGGACTCAAATGCAGCAGTTTTAGGTTGAGGGGTCTACACGGGCCTGCC
ACAGGAACCCAAGGATGCAGCTTTGCAAAGTATTGCTAA

> FE200082.Cphx2.3UTR

GTATCACAGAACCATAGCCACAGGTCAAGCCCACAGTAACCCACACAGCAGGCCAAA
GCAAAGCCGTCTCCAGAAGCCTCCACATAAAAAATAAAAATAAAAATAAGTAAATAA
ATAAATAAAAATAAATTTTTTTAAAAAAGCCAGATGAGGTGAGGTTGTTTGAGCCTAA
GGGTTCATGACGAGAGCCTGGGCAACGTGGTGAGACCCCATCTCTAAAAAAAAAAAA
AAACTTTTAAAAAAAATTAGCTGTGTGTGATAGTGAGCTCCTGTGGTACCAGCTACTCA
GGAAGCTGGTTCAGGAGGATCACTTGAGCCCAGGAGTTCAAGACCAGCCTGGGAAAC
ATTTCTCAAAAAAAAAAAAAAAAAAAACATTTAAGCCAGGCACAATGGCATGTGCCTGT

AGTCCCATTTAACTGGGAGGCTGAGATGGAAGTATCACTTGAGCCCGGGAGGTGGAGG
CTGCAGTGAGCTGTGATGGTTCCACTATATTCCGGCCTG

NOBOX (FE518599 chr7:144100745-144100865, -)

> FE518599.Nobox.5'UTR

CGCGGCGGCGGGAGTCCAGGCC

> FE518599.Nobox.CDS

ATGGAACCCACAGAGAATCCCTGCCAGGGACCGCTGGATCAGCTGGAGGAGCTAGAG
AAGATATTCCAAGAAGACCACTATCCTGACAGTGATAAACGCCGAGAGATTGCCCAGA
CGGTGGGGGTGACCCCCCAGCGCATCATGGTAAAGGGGGCCGGCTCACTGGTGGCAGG
GTGGAGTGGCGGAGGGCCCACCATTGAAACACTCGAATTGCAGAGTGAGCGCTCAGCG
GTAGCCTGGGTGTGGTTCCAGAATCGCCGGGCCAAGTGGCGAAAAATGGAGAAACTG
AATGGGAAAGAAAGCAAGGACAATCCTGCAGCCCCTGGCCCTGCCAGCAGTCAATGC
AGCTCTGCAGCTGAGATCCTACCTGCTGTGCCCATGGAGCCAAAGCCTGACCCTTTCCC
TCAGGAGTCCCCTCTGGATACCTTTCCAGAGCCCCCCATGCTGCTGACTTCTGACCAGA
CTTTGGCCCCCACCCAACCCAGTGAGGGTGCTCAGAGGGTGGTGACCCCCCCACTCTTC
AGCCCCCCACCTGTGCGAAGGGCCGATCTTCCTTTCCCCCTTGGCCCTGTCCACACCCC
CCAACTGATGCCACTGCTGATGGATGTTGCTGGCAGTGACAGCAGCCACAAGGACGGC
CCCTGTGGGTCCTGGGGGACAAGCATCACCCTGCCACCCCCCTGTTCATATTTGGAGGA
GCTGGAGCCCCAGGATTACCAACAGAGCAACCAGCCAGGACCCTTCCAGTTCTCCCAG
GCTCCACAGCCCCCGCTTTTCCAGTCCCCTCAGCCCAAGTTGCCCTACCTCCCCACTTTC
CCCTTCTCCATGCCCAGTTCACTGACGCTTCCACCGCCCGAAGACTCTCTCTTTATGTTT
CCCTGTGGCCCCAGCGGGGGCACATCGCAGGGCTATTGCCCAGGTGCCTCCTCAGGAC
AGATCCTGATGCAACCACCTGCTGGGAATATAGGTACAGCCTCCTGGAGTGACCCCTG
TTTGCCAGAGCTGCCCTTCCCTGGTCCGTTCTGCCCACAAGCTCTGGGGCATCCCCCAG
GAGGGGATGGCTACTTTCCTGATCTATTTCCAACTCCCTGCCCCCAGGCTCTGGGCAGG
CAGCCTTCGTCAGCTCTCTCATGGATGCCTGAAGGGGCCAGACCAGGGACTGGGCCCT
TACTCAGCAAGGCAAAAGAGGAACCACCAGCTGCTTCCCTGGATCAGCCCTCAGCACT
GGAGGAGGCCAGAGGGGATGACAAGAATAGCCATGTCCCCTAG

> FE518599.Nobox.3'UTR

TTGGGGGGTCAAAGAGTGAAAAGAGGCTGCTTGGGTGGGATAGGATTGGGCTCGCTGA
GAGGACAGGGGTTGGGGGTTTGGGAAAGAGTGTGGACTCTGTAGACTGAGGGGAGAG
TTAGACGTTTCCCCTGTGTAGGCTCGTCTTGTTGATATGAGAAGGAAGAAAGAGGGCT
GGCGCTTCCCTTTTCTTCACACTCTGTGTGGGGGTCCTGAAGGAAGCAGGTGGCCTGTG
TTACTTCTGAATGTTGTATCAGGTGTTTTTGTTTGTTTCCTATCCTTGCTTGACAAATGA
GCTG

Supplementary text 2

UCSC genome browser tracks for novel PRD-like homeobox genes

ARGFX

track name="ARGFX" description="ARGFX clones and the potential ORFs in human preimplantation development" visibility=2 itemRgb="On" colorByStrand="0,128,255 255,128,0"

chr3 121286806 121305447 ARGFX_D3_01 1000 +
121286832 121305444 0 3 54,149,579, 0,16957,18062

chr3 121286806 121305447 ARGFX_D3_02 1000 +
121286832 121305444 0 4 54,129,149,579,
0,2728,16957,18062

chr3 121286806 121305447 ARGFX_D3_03 1000 +
121286832 121289610 0 6 54,115,117,117,149,579,
0,2742,8824,12019,16957,18062

chr3 121286806 121305447 ARGFX_D3_04 1000 +
121286832 121289610 0 5 54,115,117,149,579,
0,2742,8824,16957,18062

chr3 121286806 121305447 ARGFX_D3_05 1000 +
121286832 121289610 0 4 54,115,117,579,
0,2742,8824,18062

chr3 121286806 121305447 ARGFX_D3_06 1000 +
121286832 121289610 0 5 54,115,117,149,579,
0,2742,12019,16975,18062

CPHX1

track name="CPHX1" description="CPHX1 clone and the potential ORF in human preimplantation development" visibility=2 itemRgb="On" colorByStrand="0,128,255 255,128,0"

chr16 75748028 75760388 CPHX1_D3_01 1000 -
75748124 75760340 0 4 1092,194,43,29,
0,4134,12287,12331

CPHX2

track name="CPHX2" description="CPHX2 clones and the potential ORF in human preimplantation development" visibility=2 itemRgb="On" colorByStrand="0,128,255 255,128,0"

chr16 75694124 75710907 CPHX2_D3_01 1000 -
75694154 75710897 0 4 821,202,194,23,
0,821,9136,16760

chr16 75694124 75710907 CPHX2_D3_02 1000 -
75694776 75710897 0 3 916,194,23, 0,9136,16760

chr16 75694124 75710907 CPHX2_D3_03 1000 -
75694154 75694966 0 2 1023,23, 0,16760

DPRX

track name="DPRX" description="DPRX clone and the potential ORF in human preimplantation development" visibility=2 itemRgb="On" colorByStrand="0,128,255 255,128,0"

chr19 54135319 54140277 DPRX-D3_01 1000 +
54135360 54140239 0 3 69,155,428, 0,2465,4530

chr19 54135319 54140277 DPRX-D3_02 1000 +
54135360 54140091 0 2 69,428
0,4530

DUXA

track name="DUXA" description="DUXA clone and the potential ORF in human preimplantation development" visibility=2 itemRgb="On" colorByStrand="0,128,255 255,128,0"

chr19 57665723 57678838 DUXA_D3_01 1000 -
57665779 57678808 0 6 127,106,146,112,155,52,
0,911,3972,4811,6287,13063

DUXB

track name="DUXB" description="DUXB clones and the potential ORF in human preimplantation development" visibility=2 itemRgb="On" colorByStrand="0,128,255 255,128,0"

chr16 75727792 75735357 DUXB_D3_01 1000 -
75727829 75735316 0 5 631,155,106,155,66,
0,2066,2943,6120,7499

chr16 75727792 75735357 DUXB_D3_02 1000 - 75727829 75728423 0 4 631,155,106,66, 0,2066,2943,7499

chr16 75727792 75735357 DUXB_D3_03 1000 - 75727829 75735316 0 3 631,155,66, 0,2066,7499

NOBOX

track name="NOBOX" description="NOBOX clone and the potential ORF in human preimplantation development" visibility=2 itemRgb="On" colorByStrand="0,128,255 255,128,0"

chr7 144094186 144100786 NOBOX_D0_01 1000 - 144094335 144100783 0 7 448,305,229,86,107,107,37, 0,1188,1856,2303,2663,3112,6563

TPRX2

track name="TPRX2" description="TPRX2 clones and the potential ORFs in human preimplantation development" visibility=2 itemRgb="On" colorByStrand="0,128,255 255,128,0"

chr19 48362491 48364978 TPRX2_D3_01 1000 + 48362503 48364766 0 3 37,170,920, 0,878,1567

chr19 48362491 48364978 TPRX2_D3_02 1000 + 48362503 48364201 0 3 37,75,920, 0,973,1567

chr19 48362491 48364978 TPRX2_D3_03 1000 + 48362503 48364818 0 3 37,170,316, 0,878,2171

TPRX1

track name="TPRX1" description="TPRX1 clone and the potential ORF in human preimplantation development" visibility=2 itemRgb="On" colorByStrand="0,128,255 255,128,0"

chr19 48304818 48307808 TPRX1_D3_01 1000 - 48305031 48307796 0 5 662,277,129,170,37, 0,1010,1290,1942,2953

Supplementary Table 1

Gene	Predicted promoters	Total number of normalized tagclusters in predicted transcript	Total number of normalized tagclusters in gene area
ARGFX	0	0	1,07
DUXA	0	0,8	0,8
DPRX	0	0	0
NOBOX	0	0	0,37
CPHX1	0	2,59	2,59
CPHX2	0	0	0
DUXB	1	35,94	35,94
TPRX2	0	0	0
OTX2	22	> 10 000	> 10 000

Supplementary Table 4

Gene	Forward Primer	Reverse Primer
ARGFX	GCCAGACCATGTTCCCAGAT	TTTGAATCGCCGGTTCCTGA
CPHX1	TCTCAGTTGCTTGCTGGTCTC	GGAAACCACCTGAAGTGCCG
CPHX2	GAGTTTCGACATGTCTTCCCAAG	GGTGAAATCAGGATAACCGTTCTC
DPRX	GAAGATGCCAGGCTCAGAGG	AAGGCTGGGGTTTGGGTATG
DUXA	GGGCAAGATCAACCTGGTGT	TGTGTAACTGAGAGGCGCTG
DUXB	GGCCAAAGAAATTGGGGTTCC	GAGTCAGATGCTGGGACTGG
GAPDH	GAGTCAACGGATTTGGTCGT	CATGGGTGGAATCATATTGG

Supplementary Table 5

Gene	Forward Primer with AscI site	Reverse Primer with PACI site
ARGFX	atatatGGCGCGCCATGAGGAACAGAATGGCCC	cgcgcgTTAATTAATCAGAGAAATCCCAAGTCTACCATAT T
CPHX1	atatatGGCGCGCCATGAATTTGGACGGCAC	cgcgcgTTAATTAATTAAAGTTGCTGCATTTGG
CPHX2	atatatGGCGCGCCATGTCTTCCCAAGCTTTC	cgcgcgTTAATTAATTAGCAATACTTTGCAAAGC
DPRX	atatatGGCGCGCCATGCCAGGCTCAGAGGATCT	cgcgcgTTAATTAATCATCTCTCTCGACTTTGGTTAGAT
DUXA	atatatGGCGCGCCATGGCCGAAGACACCTATTC	cgcgcgTTAATTAATCACCACGTTCTGGCTC
DUXB	atatatGGCGCGCCATGAATTTGGAGGGCACT	cgcgcgTTAATTAATCAGTGTGTCCCTTTGAG
NOBOX	atatatGGCGCGCCATGGAACCCACAGAGAATC	cgcgcgTTAATTAACTAGGGGACATGGCTATTC
OTX2	atatatGGCGCGCCATGATGTCTTATCTTAAGCAACCG	cgcgcgTTAATTAATCACAAAACCTGGAATTTCCA
TPRX2	atatatGGCGCGCCATGCAAGACCCTGGTC	cgcgcgTTAATTAACTATAAATCCAGTAATGACCTG
mCherry	atatatGGCGCGCCATGGTGAGCAAGGGCGAGGAG	cgcgcgTTAATTAATACTTGTACAGCTCGTCCATG

Suuplementary Figure 1

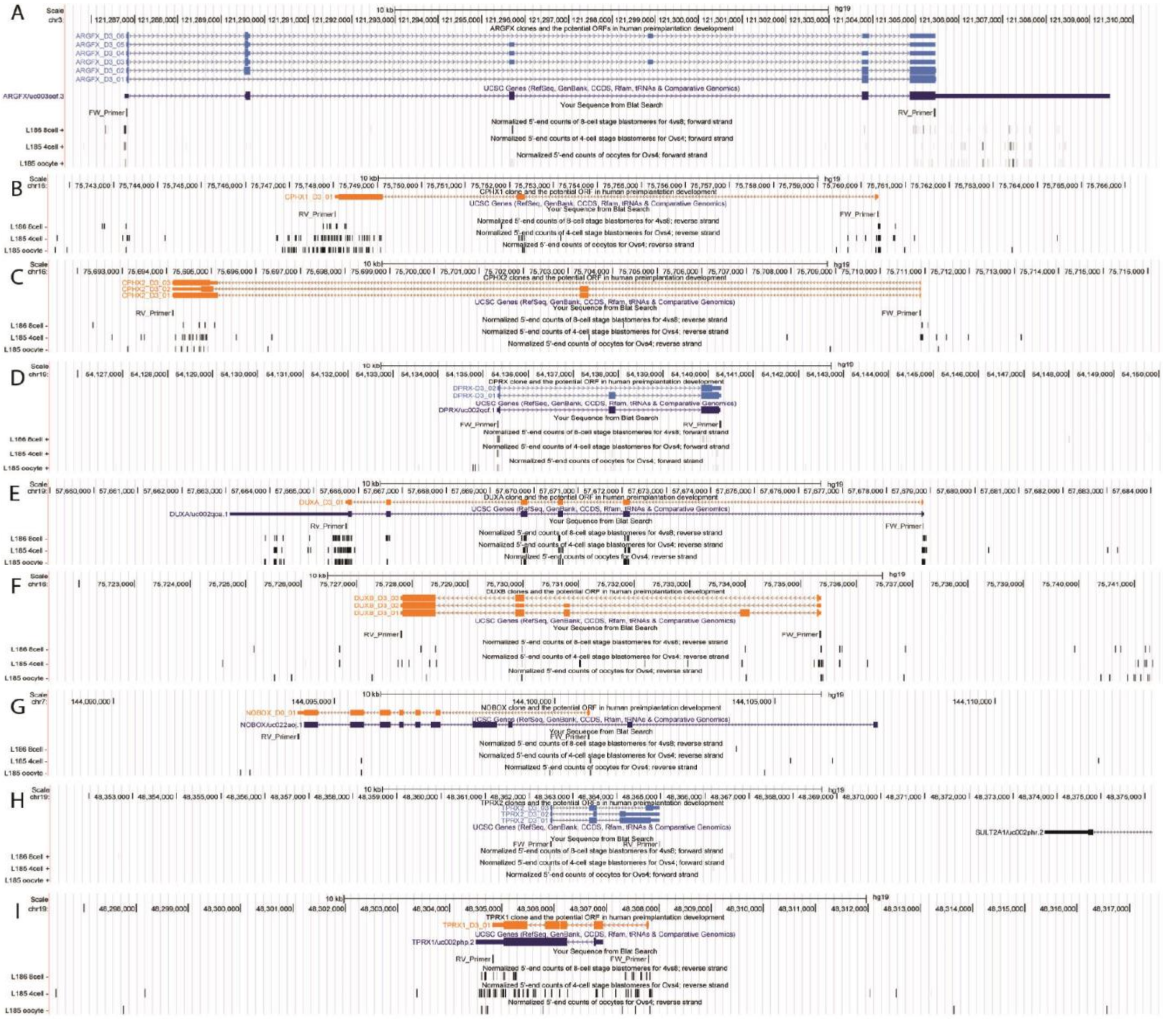

Supplementary Figure 2

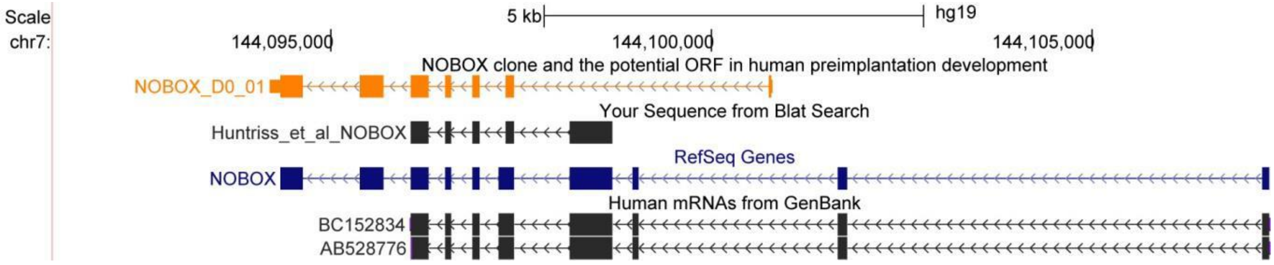

Supplementary Figure 3

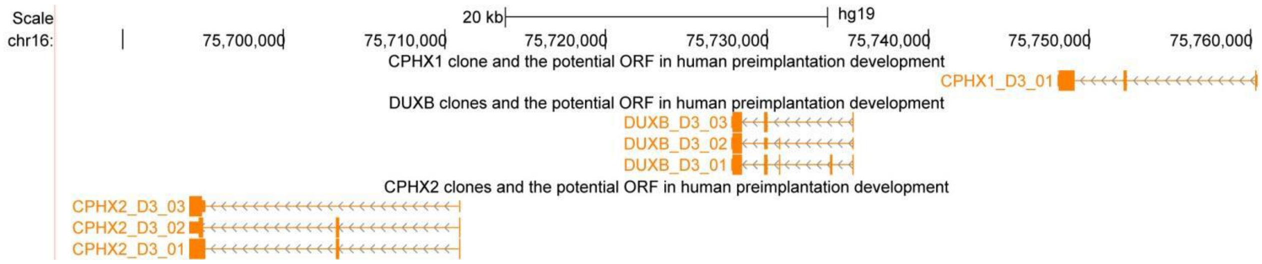

Supplementary Figure 4

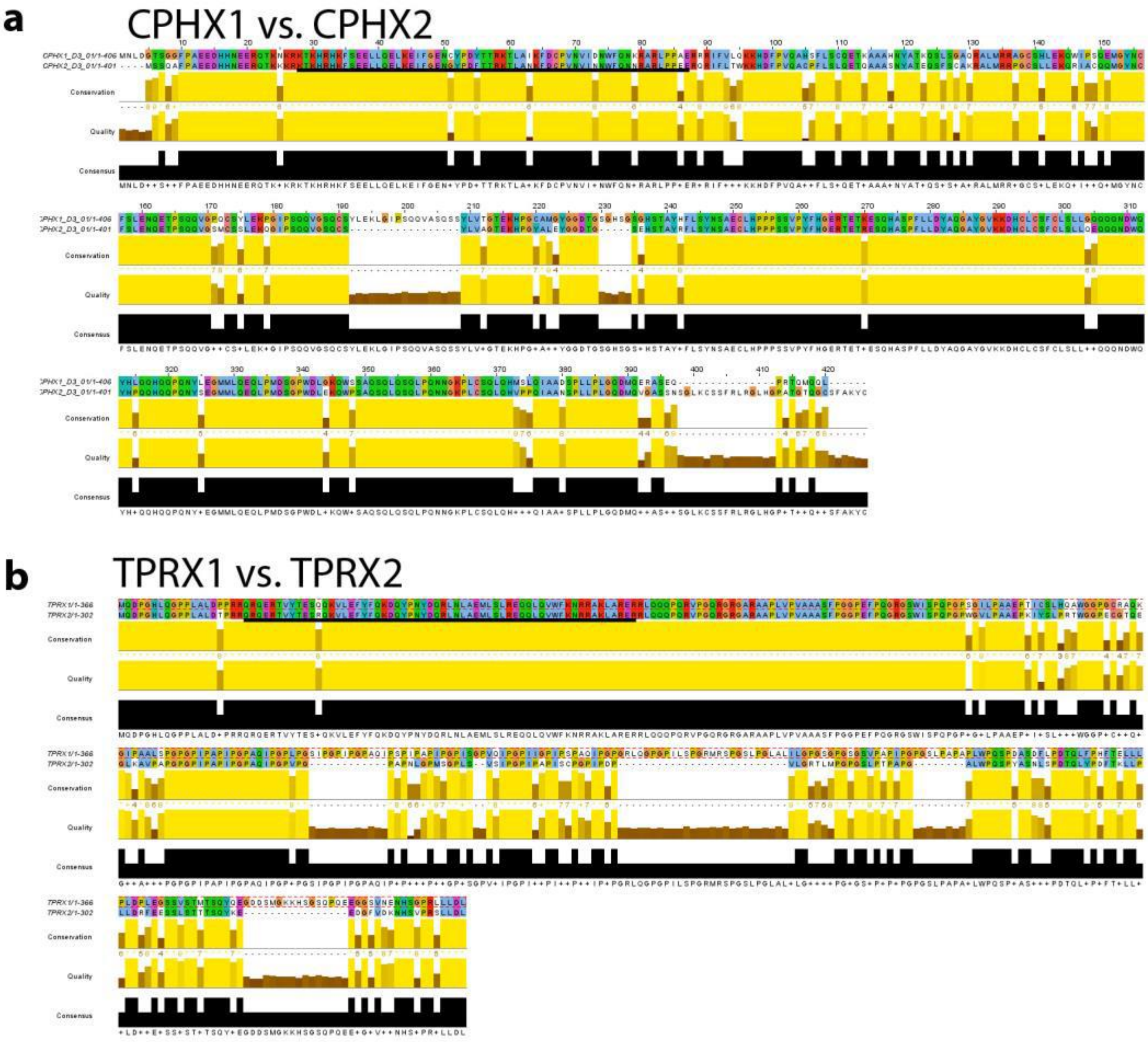

Supplementary Figure 5

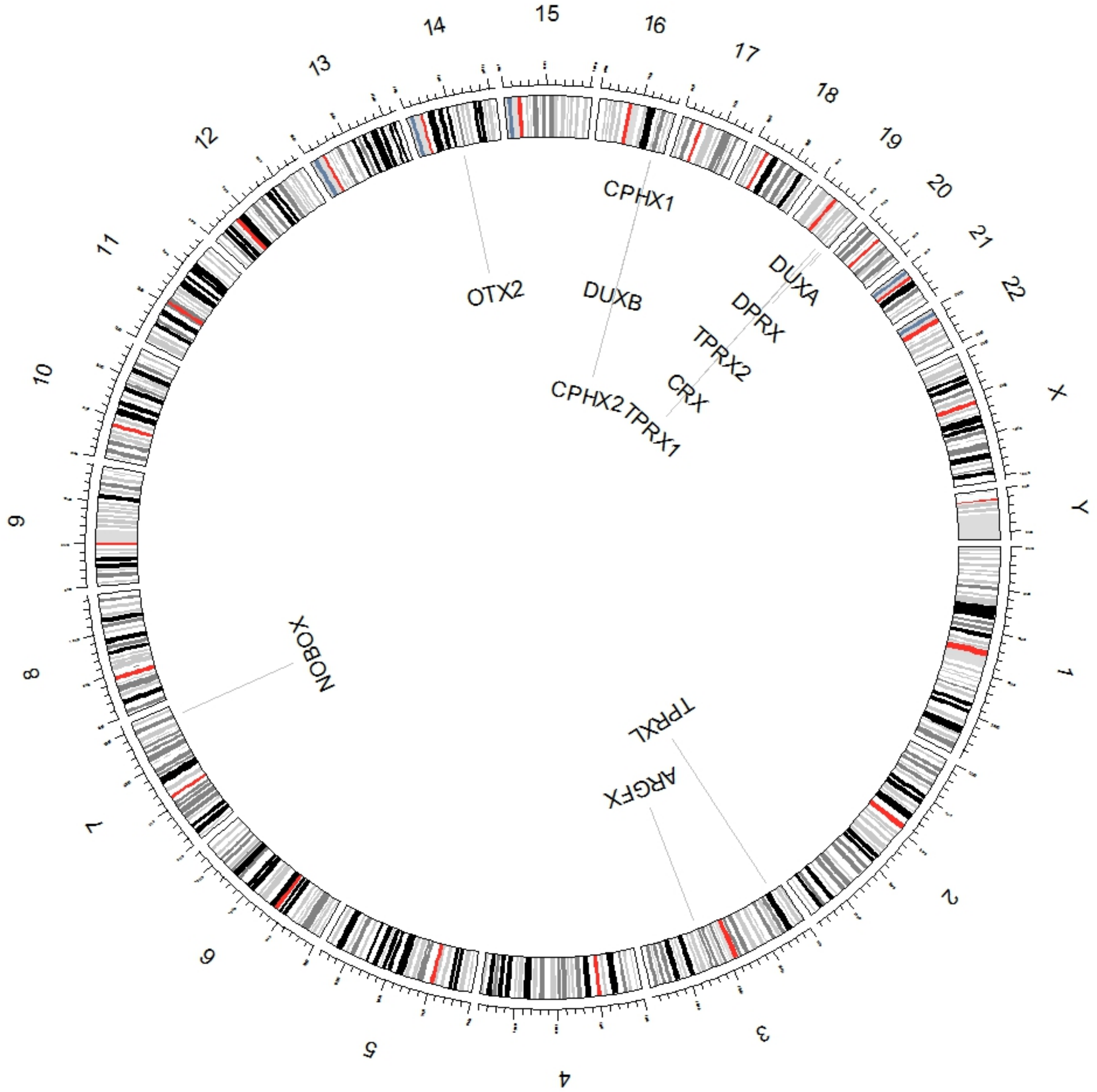

Supplementary Figure 6

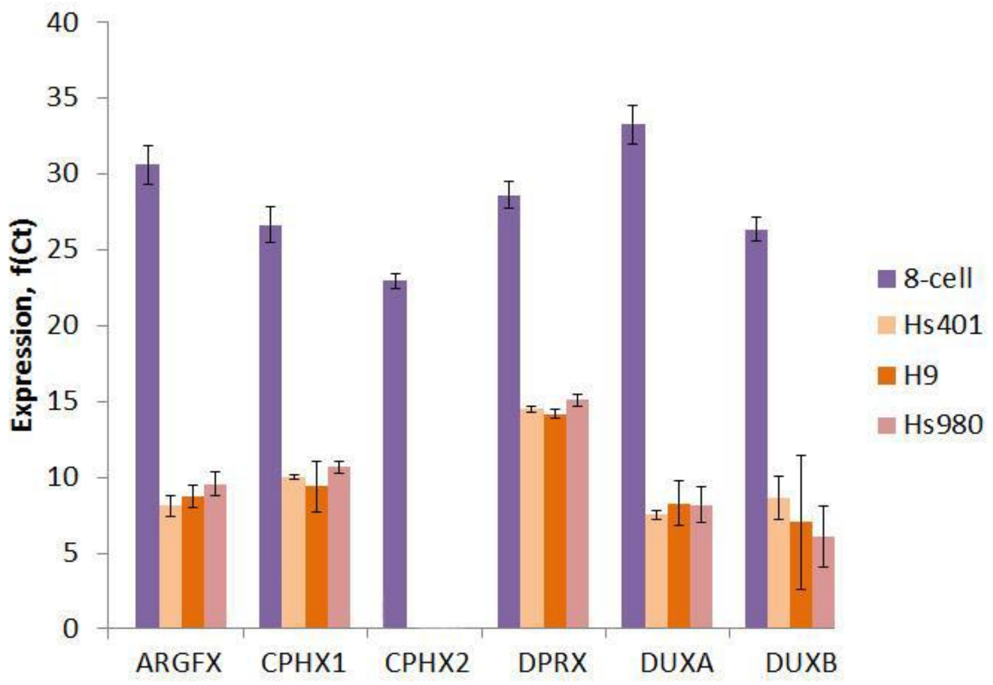

Supplementary Figure 7

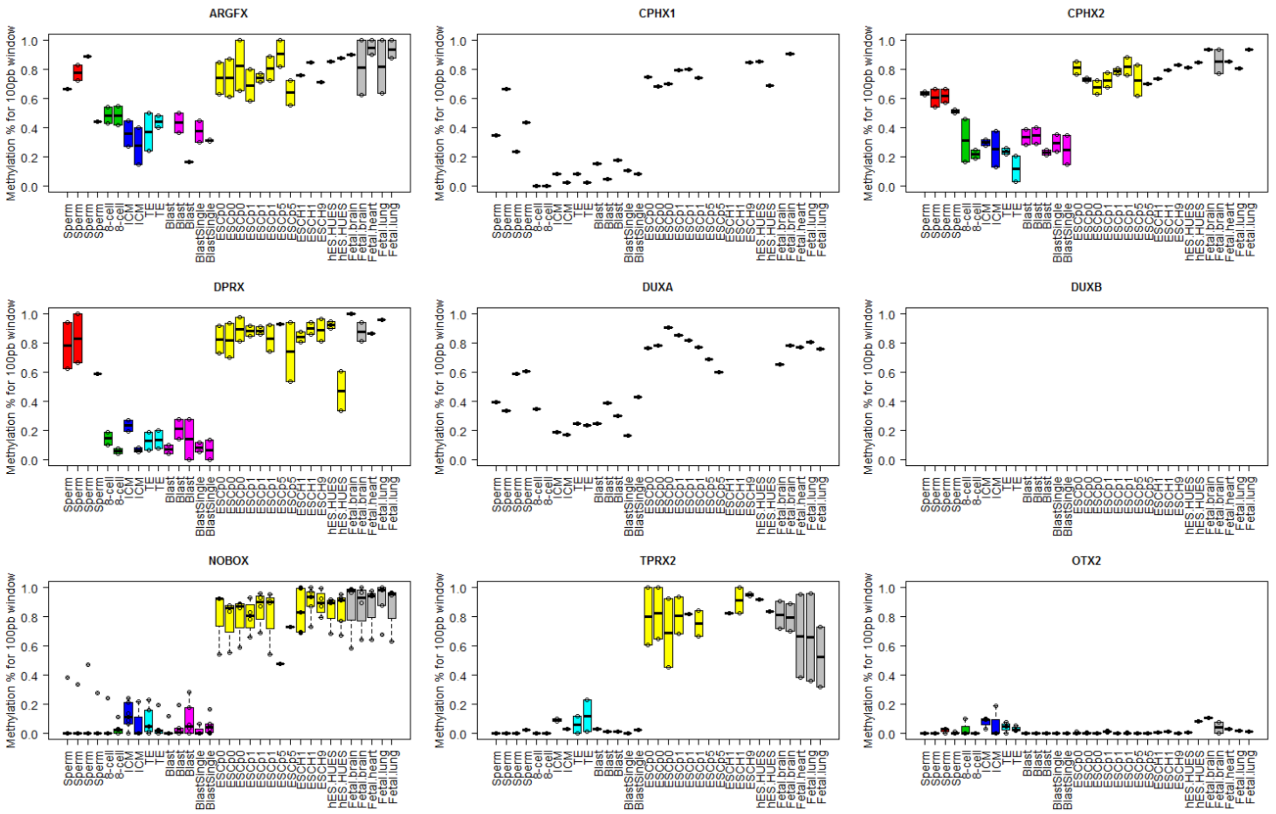

Supplementary Figure 8

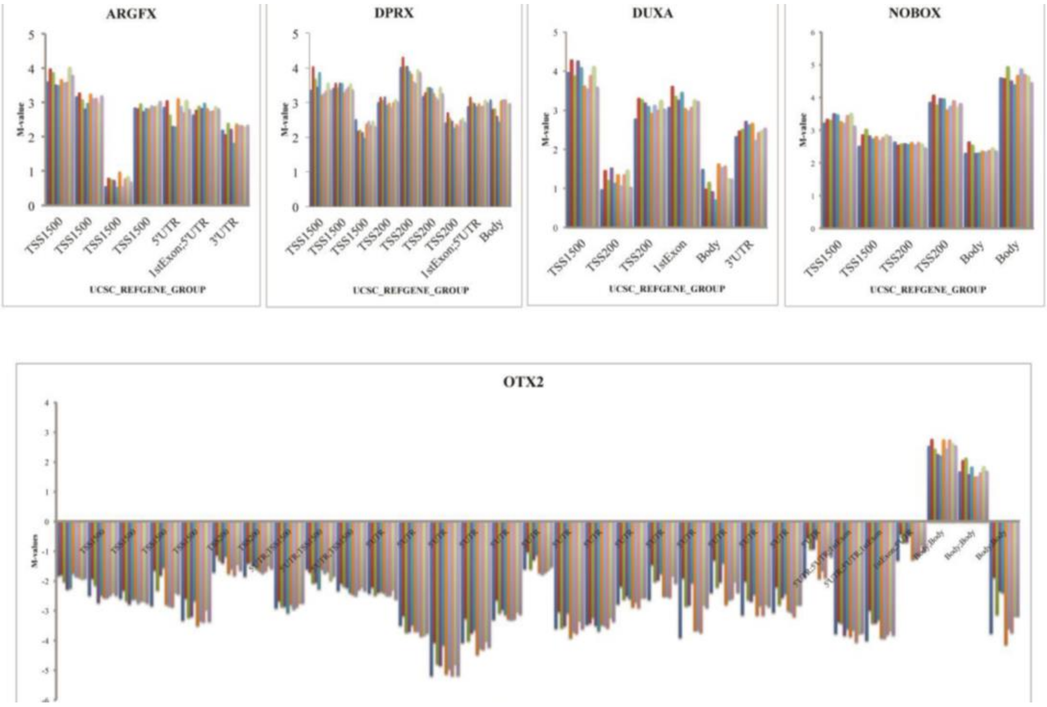

Supplementary Figure 9

a

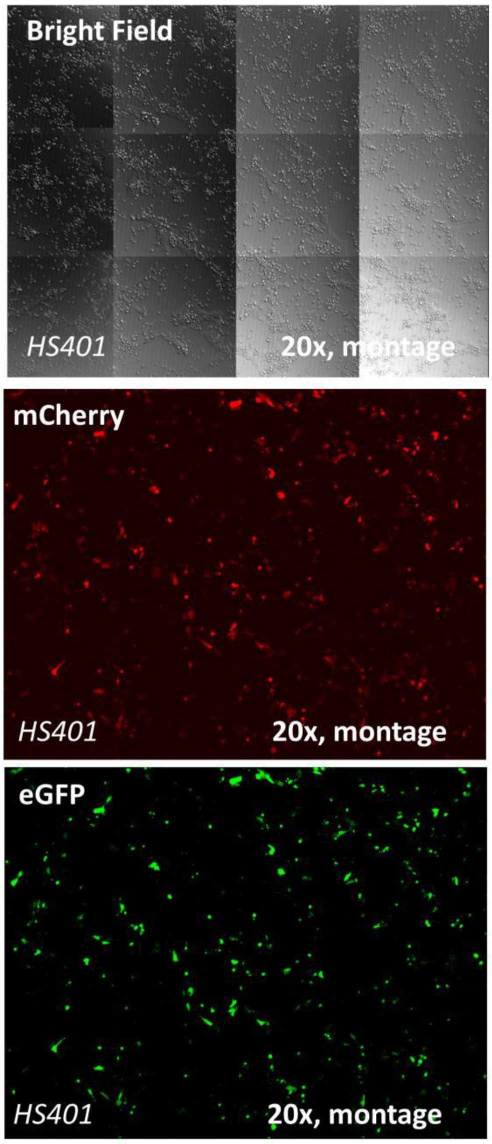

b

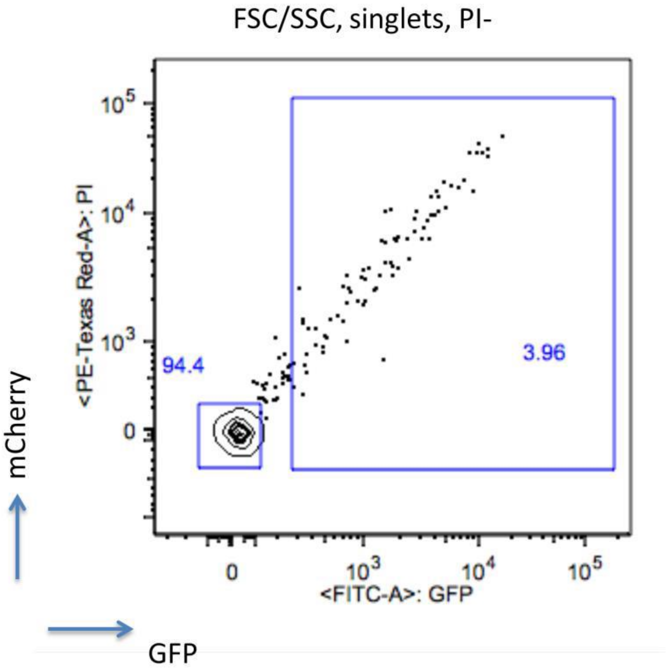

c

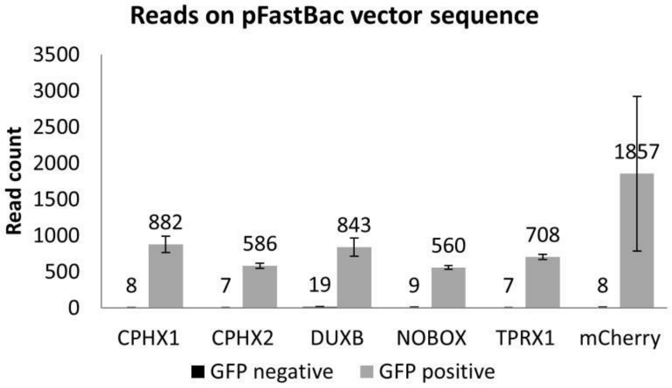

Supplementary Figure 10

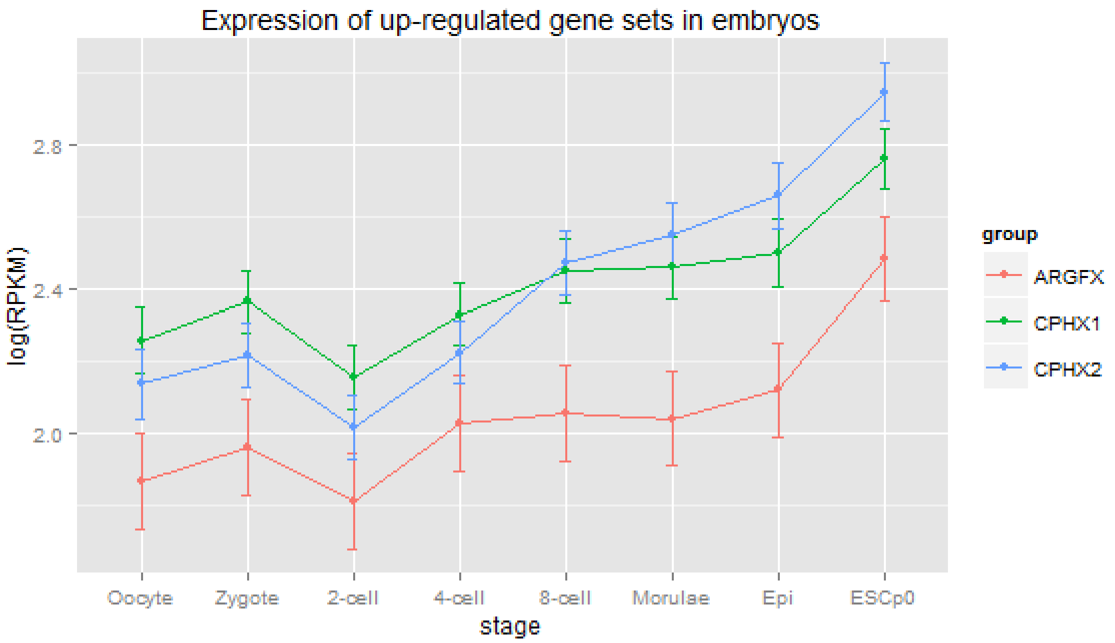

Supplementary Figure 11

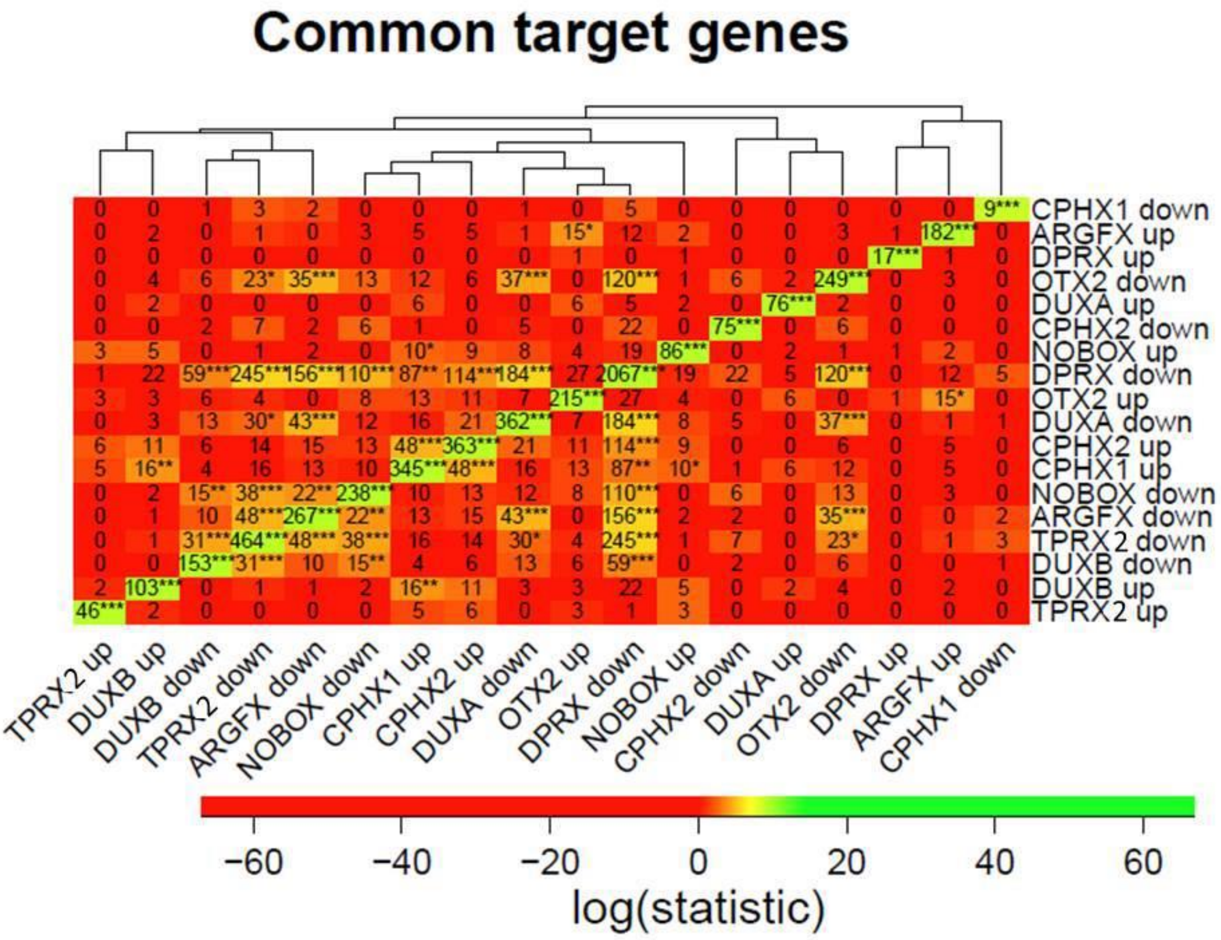

Supplementary Figure 12

a

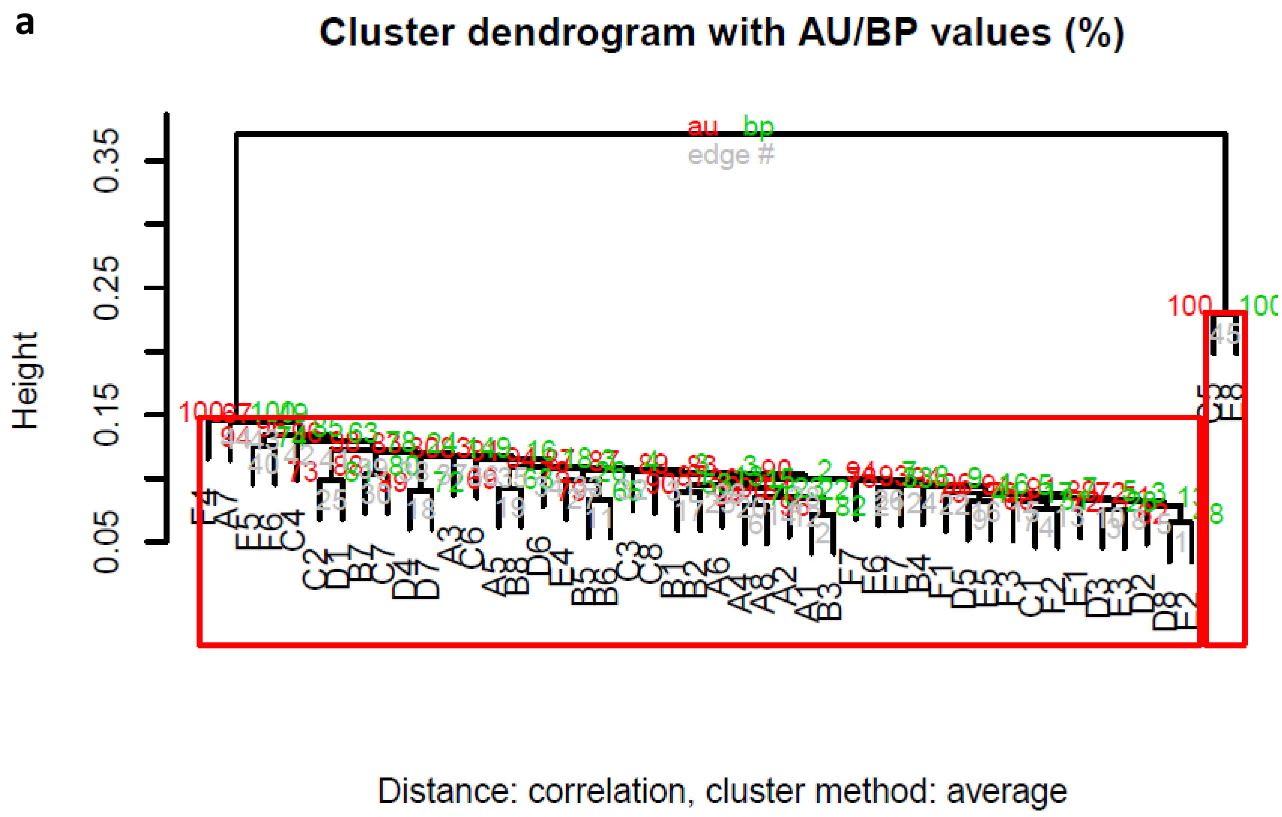

b

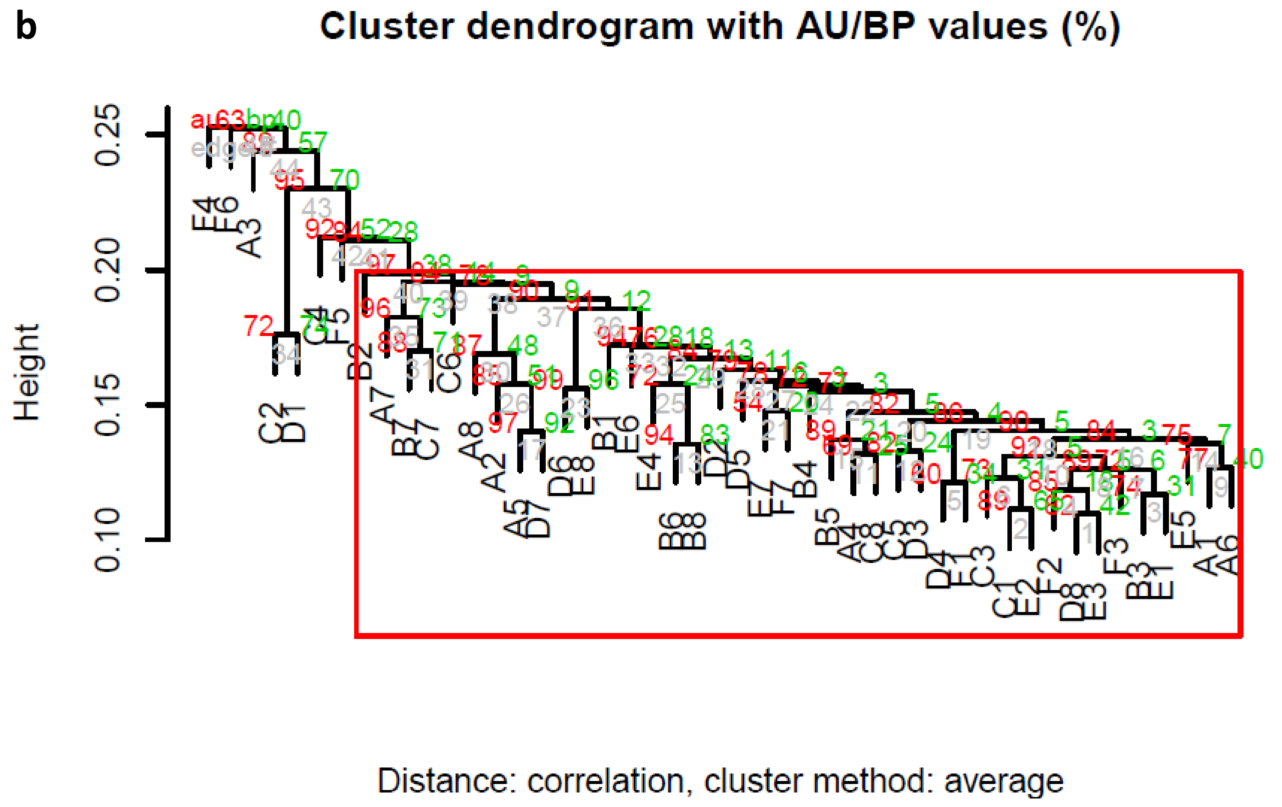

Supplement: Supplementary Information [file srep28995-s1.pdf]
